# Development of Liposomal Ciprofloxacin to Treat Lung Infections

**DOI:** 10.3390/pharmaceutics8010006

**Published:** 2016-03-01

**Authors:** David Cipolla, Jim Blanchard, Igor Gonda

**Affiliations:** Aradigm Corp., Hayward, CA 94545, USA; blanchardj@aradigm.com (J.B.); gondai@aradigm.com (I.G.)

**Keywords:** liposomes, formulation, ciprofloxacin, lung infection, cystic fibrosis, bronchiectasis, non-tuberculous mycobacteria, inhalation delivery

## Abstract

Except for management of *Pseudomonas aeruginosa* (PA) in cystic fibrosis, there are no approved inhaled antibiotic treatments for any other diseases or for infections from other pathogenic microorganisms such as tuberculosis, non-tuberculous mycobacteria, fungal infections or potential inhaled biowarfare agents including *Francisella tularensis*, *Yersinia pestis* and *Coxiella burnetii* (which cause pneumonic tularemia, plague and Q fever, respectively). Delivery of an antibiotic formulation via the inhalation route has the potential to provide high concentrations at the site of infection with reduced systemic exposure to limit side effects. A liposomal formulation may improve tolerability, increase compliance by reducing the dosing frequency, and enhance penetration of biofilms and treatment of intracellular infections. Two liposomal ciprofloxacin formulations (Lipoquin^®^ and Pulmaquin^®^) that are in development by Aradigm Corporation are described here.

## 1. Introduction

### 1.1. Rationale for Inhaled Antibiotics

The rationale to deliver antibiotics as inhaled aerosols to treat lung infections is straightforward: lower doses can be used to achieve high local antibiotic concentrations in the lung while reducing systemic exposure and thus the potential for side effects [1]. In contrast, the traditional approach of using oral or intravenous (IV) injections of antibiotics requires administration of much higher systemic doses which may still provide inadequate drug concentrations in the lung to combat the infection. For example, when the aminoglycoside antibiotic amikacin is given by inhalation, the maximum drug levels in the bronchial secretions exceed those in serum by a factor of more than 1000; in contrast, IV administration resulted in three-fold higher drug concentrations in serum than in the bronchial secretions [2]. Thus, targeting amikacin to the lung by inhalation leads to a >3000-fold differential in the lung to serum drug concentration relative to that of IV administration. Given that amikacin exhibits concentration-dependent killing, for which optimal antimicrobial effect requires the peak drug concentration to exceed the minimum inhibitory concentration (MIC) of the infectious agent by at least a factor of 10 (peak/MIC > 10) [3], inhalation targets the lung with peak/MIC ratios that vastly exceed 10 while systemic delivery does not. Amikacin, similar to other aminoglycosides such as gentamicin and tobramycin, causes nephrotoxicity and ototoxicity [4] and, therefore, it is desirable to keep the systemic exposure as low as possible.

**Figure 1:**
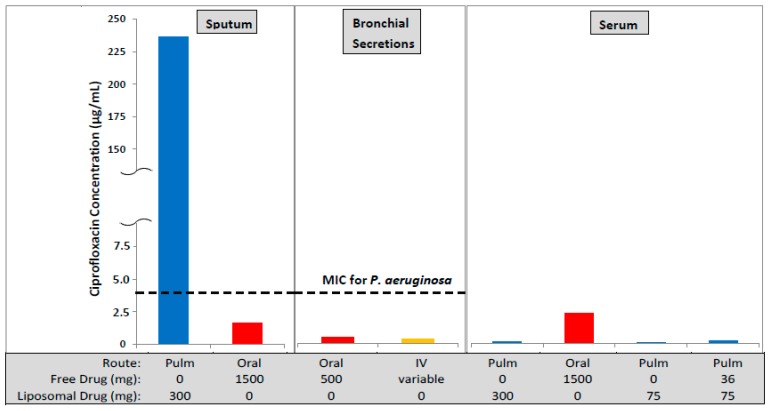
The concentration of ciprofloxacin in lung sputum (*C*_max_), bronchial secretions (mean concentration), and serum (*C*_max_) following inhaled (Pulm) [9,10], oral [11] or intravenous (IV) [11] administration. The oral and IV formulations contained unencapsulated (free) ciprofloxacin while the inhaled formulations contained liposomal ciprofloxacin (Lipoquin) or combinations of free and liposomal ciprofloxacin. The oral dose, even at twice the maximum labeled dose of 750 mg, leads to a maximum drug concentration in the sputum lower than the MIC for *Pseudomonas aeruginosa* in the biofilm of 4 µg/mL [12]. In contrast, the maximum drug concentration in the sputum by inhalation exceeds the MIC by more than 50-fold and the mean concentration over the 24 h dosing period exceeds the MIC by 20-fold [9]. However, the peak serum drug concentration following inhalation is only a fraction (~3%–5%) of that for oral dosing thus reducing the potential for systemic side effects.

A similar rationale exists for ciprofloxacin, a fluoroquinolone antibiotic, with the ability to achieve higher drug concentrations in the lung and lower systemic drug concentrations for inhaled compared to oral or IV administration of ciprofloxacin (Figure 1). In fact, the use of IV ciprofloxacin to treat lower respiratory tract infections [5] or severe pneumonia [6] was often ineffective for patients colonized with *Pseudomonas aeruginosa* (PA): observations included failure to eradicate the organism [5], persistence of infection and emergence of resistance [5,6]. The publications concluded that intravenous doses of 300 (or as high as 400) mg every 12 h may be ineffective against PA lung infections due to inadequate drug concentrations in the lung [6]. In patients with severe pneumonia colonized with PA, even higher doses of IV ciprofloxacin (400 mg every 8 h) failed to achieve eradication and led to the emergence of resistance [5]. Thus, an inhaled ciprofloxacin product which achieves higher drug concentrations in the lung may be more effective and reduce the potential for development of resistance.

Fluoroquinolones, like aminoglycosides, are generally thought to exhibit concentration-dependent antimicrobial effect instead of time-dependent killing for which effectiveness is related to the time the drug concentration remains above the MIC [3]. However, ciprofloxacin appears to operate by a blending of both the time-dependent and concentration-dependent elements [7]. For ciprofloxacin, the ratio of the area under the concentration time curve (AUC) divided by the MIC appears to be the best predictor of antimicrobial effect with values of AUC/MIC > 125 correlating with better cures [7,8]. Ciprofloxacin, like most small molecules, is rapidly absorbed from the lung [9]. While it is relatively easy to measure the AUC in serum by taking serial blood samples, when the site of infection is in the lung, the drug concentration in the lung is a more relevant parameter. However, it is difficult to serially sample bronchial secretions or sputum from the lung to measure the ciprofloxacin concentrations over time. In practice it may be possible to obtain a few (e.g., three) sputum samples over a 24-hour period from a small number of productive patients to verify that the drug concentration remains above the MIC until the next dosing event. Thus, an inhaled ciprofloxacin formulation which provides a sustained release profile in the lung with drug concentrations above the MIC for a longer period of time may have superior antimicrobial effect to one which requires frequent administration to ensure that the drug concentration remains above the MIC.

### 1.2. Opportunities to Treat Lung Infections

Inhaled antibiotics have been extensively utilized in the treatment of PA infections in cystic fibrosis (CF) following the approval of the first inhaled antibiotic, tobramycin, in 1997 [1]. In CF, inhaled antibiotics delay the onset of chronic colonization with PA, improve the quality of life (QoL), and reduce the rate of decline of lung function, exacerbations and hospital admissions [1]. The lifespan of CF patients has increased by more than 10 years during the past two decades and a contributing factor is that inhaled antibiotic therapy has become a standard of treatment [1]. The currently approved antibiotics include aminoglycosides (tobramycin) and monobactam (aztreonam) in the US, as well as polymixins (colistin and the related colomycin) in Europe [1]. However, some patients may poorly tolerate the existing inhaled antibiotic products or find that their infections are suboptimally treated by them [2]. Thus, there is a need for improved formulations or different classes of antibiotics that are not yet approved for inhalation (e.g., quinolones).

There are a number of other indications associated with PA lung infections for which no inhaled antibiotics are approved including non-CF bronchiectasis (NCFB) [13] and COPD [14]. In NCFB, like in CF, chronic colonization with PA results in worsening of disease: lower QoL, more rapid decline in lung function and increased mortality [13,15]. Clinical trials with the antibiotics already marketed for CF, tobramycin [16], aztreonam [17] and colistin [18], all failed to demonstrate efficacy in NCFB. All three were effective at reducing PA levels in sputum, and showed some evidence for improvements in QoL, but failed to improve lung function or reduce hospitalizations or the time to the first pulmonary exacerbation or number of such exacerbations [16,17,18]. These antibiotic formulations often required pre-treatment with bronchodilators due to their bronchoconstrictive properties but were often still not well tolerated and there was evidence of increases in the number of respiratory adverse events [16,17,18,19,20]. Better tolerability may enable an inhaled antibiotic to achieve clinical benefit in NCFB as was apparent for a liposomal formulation of ciprofloxacin tested in a phase 2 trial [21]. Not only was the PA sputum density reduced but there was a reduction in pulmonary adverse events and an improvement over time of first pulmonary exacerbation [21].

The incidence of lung infections associated with non-tuberculous mycobacteria (NTM) is increasing and there are limitations with the current treatment options, especially for those colonized with *M. avium* and *M. abscessus* [22]. NTM infections can give rise to NCFB and are often found in patients with emphysema, NCFB and CF. NTM can exist in various forms within the lung, both dormant (sessile) and replicating, and found in the airway fluid, biofilm and within alveolar macrophages. These factors make it challenging to eradicate NTM infections in the lung. A successful therapy should achieve effective concentrations of drug in the lung fluid, sputum, within biofilm, and intracellularly. To that end, inhaled formulations of amikacin [23,24,25] and ciprofloxacin [26,27,28] are using liposomes that are able to penetrate sputum and biofilm [29] and be phagocytosed by macrophages that harbor NTM infections. The promise of these more sophisticated antibiotic formulations is to improve clinical outcome for patients with NTM lung infections.

There are a number of other severe infections including melioidosis, which is endemic to South East Asia, and those which may arise due to acts of bioterrorism such as pneumonic plague, tularemia, and anthrax, which also do not have approved inhaled antibiotic therapies for prevention or treatment. Q-fever, although not lethal, is highly infectious and there have been sporadic outbreaks of this temporary debilitating disease. Thus, there remains a need for additional inhaled antibiotics with improved properties.

### 1.3. Rationale for Using a Liposomal Formulation of Ciprofloxacin

The properties of an inhaled antibiotic can be modified by use of formulation agents to improve tolerability or to modify the pharmacokinetic profile [30,31]. Improved tolerability may not only lead to improved compliance, but may also reduce the incidence of respiratory side effects; both factors which may contribute to achieving clinical efficacy. Most inhaled antibiotics have a short half-life in the lung due to rapid systemic absorption and, thus, require two (for tobramycin) or three (for aztreonam) administrations each day to ensure that drug concentrations in the lung remain adequately high [1]. High drug concentrations are essential to achieve pharmacologic effect as well as to reduce the potential for development of drug resistance [5,6]. Thus, the use of a formulation agent to extend the residence time of the antibiotic in the lung may allow for a reduction in the dosing frequency while still continuously maintaining high drug concentrations in the lung—above the MIC—a situation which may not be achieved for the current inhaled antibiotic paradigm, especially if patients are non-compliant with the multiple daily administration events.

Liposomes, in addition to providing a slow release of the antibiotic, can also deliver the drug to macrophages via phagocytosis, and liposomal formulations of antibiotics may thus improve treatment of intracellular infections such as NTM [23,24,25,26,27,28]. In addition, liposomes can mask the taste of bitter compounds and reduce the irritation associated with the drug, or the pH or osmotic perturbations that may occur in the lung in response to aerosol deposition [2,32]. An important benefit of an inhaled liposome formulation is to reduce the dosing frequency due to the sustained presence of drug in the lung [30]. Two liposomal formulations of antibiotics are in late stage development: amikacin [23,24,25,29] and ciprofloxacin [9,10,33]. Amikacin is from the aminoglycoside class of antibiotics which is already represented by inhaled tobramycin approved for the treatment of CF. In contrast, there are no approved quinolone antibiotics by inhalation so an inhaled liposomal ciprofloxacin product would represent a new class of antibiotics for treatment of lung infections by inhalation. Ciprofloxacin is a well-established and extensively utilized broad-spectrum fluoroquinolone antibiotic that inhibits topoisomerase II and IV, which are enzymes required for bacterial replication, transcription, repair, and recombination. Thus, an inhaled liposomal formulation of ciprofloxacin may be suitable for treating lung infections in CF, as well as in the other indications described above for which no inhaled antibiotics are approved. As ciprofloxacin acts by a different mechanism of action from other classes of antibiotics, it can be used in combination with them or when the other types of drugs are ineffective or cause unacceptable adverse reactions.

## 2. Pharmaceutical Development of a Liposomal Formulation of Ciprofloxacin

### 2.1. Evaluation and Selection of the Composition of Liposomal Ciprofloxacin

**Figure 2:**
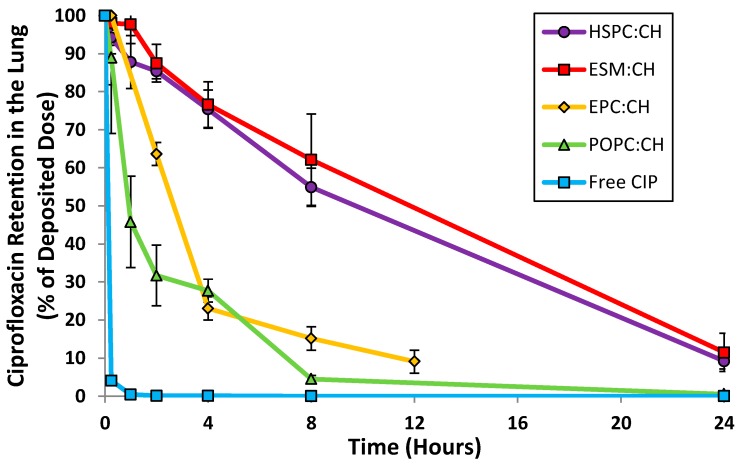
Retention of ciprofloxacin in the lung following intranasal instillation in mice (or inhalation for EPC:CH formulation). * CIP = Ciprofloxacin. CH = cholesterol. Adapted from [33,36,37].

Unilamellar liposomal formulations of ciprofloxacin were manufactured by extrusion of multilamellar liposomes through 80 nm membrane filters followed by remote loading of ciprofloxacin [33,34]. The following parameters were examined: choice of loading agent (methylamine sulfate, magnesium sulfate, ammonium sulfate and ferrous sulfate), choice of lipid (egg sphingomyelin (ESM), hydrogenated soy phosphatidylcholine (HSPC), egg phosphatidylcholine (EPC) and 1-palmitoyl-2-oleoylphosphatidylcholine (POPC)), ratio of lipid to cholesterol, and drug to lipid ratio. The various liposome compositions were evaluated by both *in vitro* and *in vivo* methods in order to select a final formulation with acceptable long term refrigerated stability, drug release consistent with once-daily dosing, retention of liposome integrity during storage and aerosol delivery, and a robust manufacturing process. The *in vivo* release profile after intratracheal instillation in mice confirmed that liposomes comprised of ESM and cholesterol, or HSPC and cholesterol, satisfied the requirement for sustained levels of ciprofloxacin in the lung for a duration of 24 h (Figure 2). In contrast, unencapsulated ciprofloxacin was rapidly absorbed within 2 h. Given the comparable *in vivo* performance for the liposomes comprised of either ESM or HSPC, HSPC was the preferred excipient based on availability of commercial GMP supplies.

Half-lives of liposomal ciprofloxacin (Lipoquin) administered to rabbits as aerosol ranged from 8.3 to 10.2 h (including in lung tissue, lavaged lung fluid and lung cells) and 13.4 h in plasma (unpublished data), consistent with the release profiles in mice. By comparison, rabbits given ciprofloxacin IV (35 mg/kg) had half-lives in serum of 1.90 h after a single dose and 2.49 h after 13 doses every 6 h [35].

**Figure 3:**
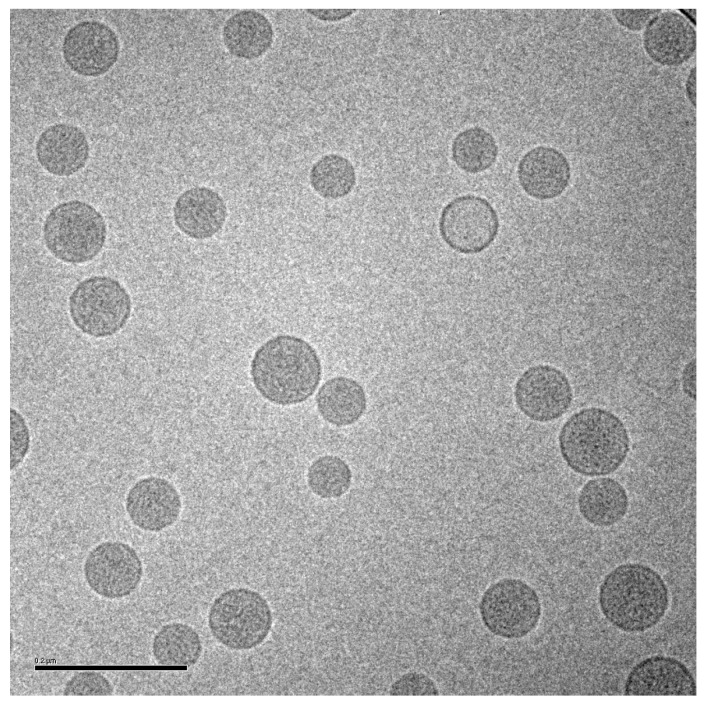
Cryo-TEM image of liposomal ciprofloxacin (Lipoquin). The scale bar is 200 nm.

Based on the *in vivo* release results in mice and rabbits, and other analyses described below, a liposomal ciprofloxacin product (termed Lipoquin^®^ (Aradigm Corp., Hayward, CA, USA)), with a bilayer composition of HSPC and cholesterol, was advanced to clinical trials [1]. These liposomes have a unilamellar morphology and are predominantly between ~50 and 100 nm in diameter [38] (Figure 3). A 1:1 mixture of liposomal ciprofloxacin (Lipoquin, 50 mg/mL) and free ciprofloxacin (FCI, 20 mg/mL) subsequently entered clinical development under the name Pulmaquin^®^ (Aradigm Corp.). Earlier prototypes of Pulmaquin combined Lipoquin with higher concentrations of FCI (up to 30 mg/mL) and are designated “Pulmaquin prototype” formulations in this article.

**Figure 4:**
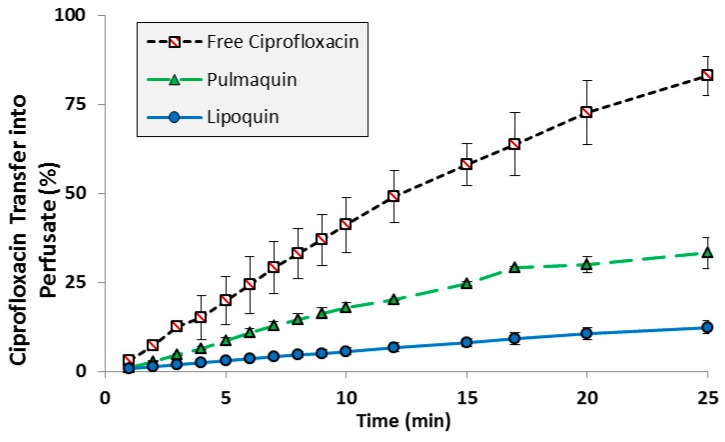
Transport of ciprofloxacin from various ciprofloxacin formulations across an Isolated Perfused Rat Lung. Adapted with permission from [39]. Copyright 2014 Elsevier.

The transport kinetics of Lipoquin, free ciprofloxacin, and Pulmaquin was investigated in an isolated perfused rat lung model [39]. The advantage of an *ex vivo* study like this is that a precise dose can be administered directly to the lung without losses in the nasal or oropharyngeal regions which occurs for inhaled or intratracheal administration. The downside of this model is that only short term studies (25 min in this case) can be performed to ensure continued tissue viability [39]. Consistent with the relative pharmacokinetics for free ciprofloxacin and Lipoquin (HSPC:CH) reported in the mice (Figure 2), Lipoquin had the slowest transport rate and free ciprofloxacin had the fastest transport rate into the perfusate in the isolated perfused rat lung (Figure 4). After 25 min, 82% ± 5.5% of the free ciprofloxacin formulation was transported into the perfusate *versus* 12.3% ± 1.8% for Lipoquin. As expected, the combination formulation (Pulmaquin) had an intermediate rate of ciprofloxacin transport into the perfusate in 25 min (33.1% ± 4.3%), presumably due to the rapid absorptive clearance of the free ciprofloxacin component. While the relative half-life of transport in this *ex vivo* model may underestimate the true half-life *in vivo*, the relative difference in transport for liposomal ciprofloxacin to free ciprofloxacin is maintained (approximately eight-fold slower transport rate for liposomal ciprofloxacin).

### 2.2. Stability of Liposomal Ciprofloxacin to the Nebulization Process

**Figure 5:**
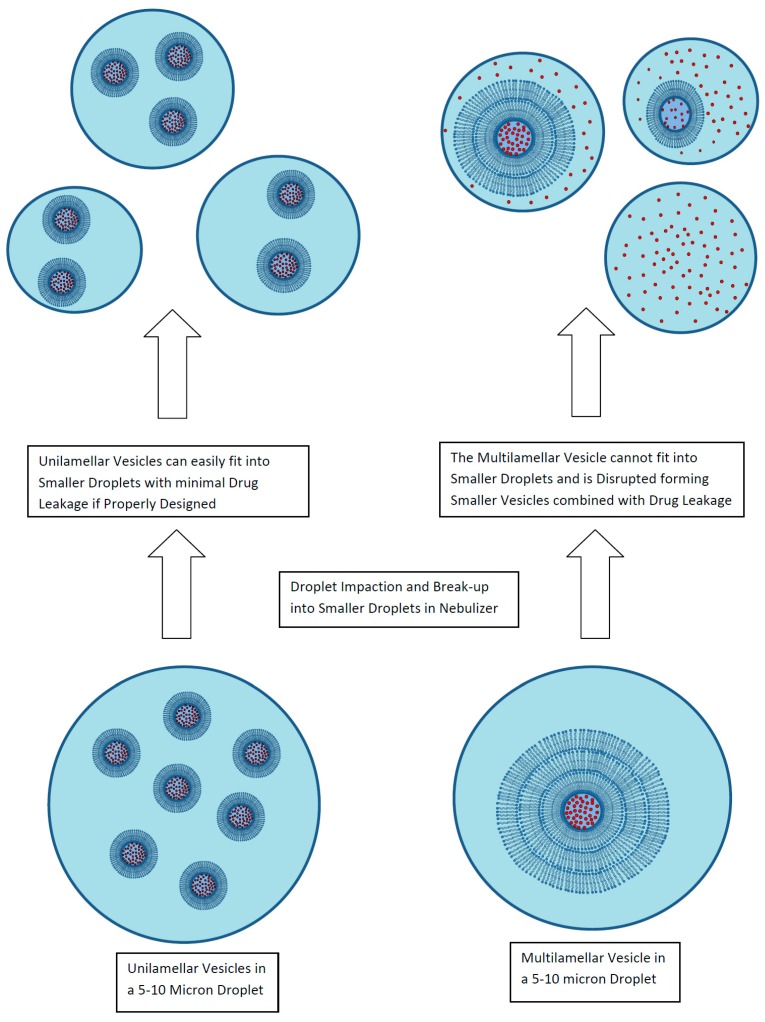
Liposome disruption during exposure to nebulization. Reprinted with permission from [30]. Copyright 2013 Future Science.

A key attribute of a liposomal formulation for inhalation is for the liposomes to be robust to the aerosol delivery process without loss of encapsulated drug, change in vesicle size, or alteration in the release properties [30]. For liposomal formulations developed in a liquid format, the primary delivery route is by nebulization, either using jet nebulizers or mesh nebulizers. In either case, the nebulization process exposes the formulation to shear and formation of extensive air–liquid interfacial surfaces (Figure 5) [30]. These processes can lead to disruption of multilamellar liposomes as well as many unilamellar liposomes including an observed 30% loss of encapsulated amikacin from the ~300 nm liposomes in Arikayce^®^ (Insmed Inc., Monmouth Junction, NJ, USA) [30,40]. To stabilize liposomes to nebulization, unilamellar liposomes of 100 nm or smaller are recommended [30]. However, the choice of drug, manufacturing method, and liposome composition all play an important role as well as the aerosolization method [30]. Liposomes composed of high phase transition temperature lipids (e.g., HSPC) incorporating cholesterol for rigidity may provide greater stability to nebulization [30].

Both Lipoquin and Pulmaquin have been evaluated for their ability to withstand the rigors of the nebulization process [41,42]. One batch of Lipoquin was placed on stability for six months at room temperature conditions and 24 months at refrigerated conditions [41]. At each stability time point, 5 mL was nebulized in a PARI LC Sprint^®^ nebulizer and the residual solution in the nebulizer, as well as the collected aerosol, were evaluated for retention of drug encapsulation (Table 1). The liposomes in the collected aerosol as well as that remaining in the nebulizer after exposure to the full nebulization process retained drug encapsulation comparable to that of the control liposomes (99%), not only right after manufacture (initial), but also after every stability time point. For subsequent batches, the aerosol characterization was performed after 24 months of refrigerated storage (Table 2). In these studies, in addition to retention of drug encapsulation, it was also confirmed that the mean vesicle size was unchanged in response to storage and nebulization. The drug recovery averaged 95% demonstrating acceptable mass balance and verifying that the experimental technique was adequate.

**Figure 6:**
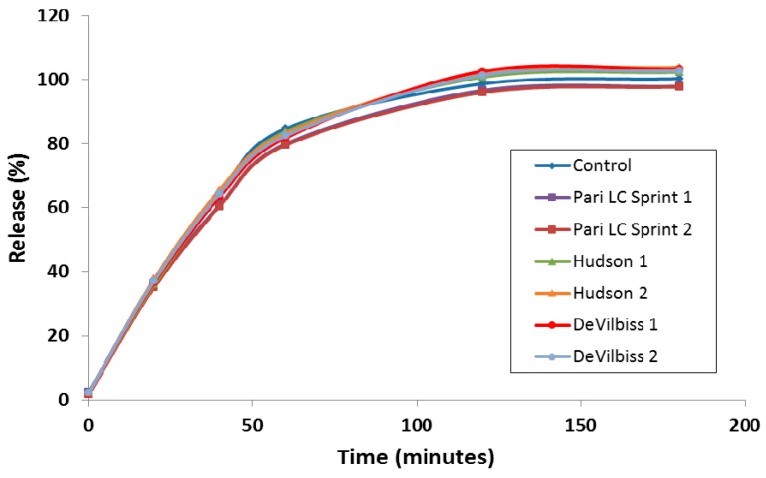
Lipoquin retains its IVR profile following nebulization using the PARI LC Sprint^®^, Hudson T Updraft II^®^ and the DeVilbiss Permaneb^®^. Two experiments were conducted for each nebulizer labeled 1 and 2. Reprinted with permission from [42]. Copyright 2013 RDD online.

A discriminating *in vitro* release (IVR) assay was developed to compare the release properties of liposomal ciprofloxacin batches in development and reduce the requirement for laborious *in vivo* studies to make development decisions [43]. For ease of execution by laboratory personnel, the IVR assay was designed for complete release of ciprofloxacin from the liposomes within a 2–4 h time frame [43], in contrast to the *in vivo* PK data which shows release over a 24 h time period (Figure 2). The IVR assay demonstrated that the release properties of Lipoquin were maintained following nebulization using three different nebulization systems (Figure 6) [42]. The PARI LC Sprint nebulizer is the one that has been used in the ongoing Lipoquin and Pulmaquin clinical programs. The other two nebulizers were selected to test the robustness of Lipoquin because they were used previously for an unrelated liposomal ciprofloxacin formulation and found to disrupt liposomal ciprofloxacin preparation causing extensive drug leakage following nebulization [44]. Lipoquin showed no change in the *in vitro* release profile following nebulization.

**Figure 7:**
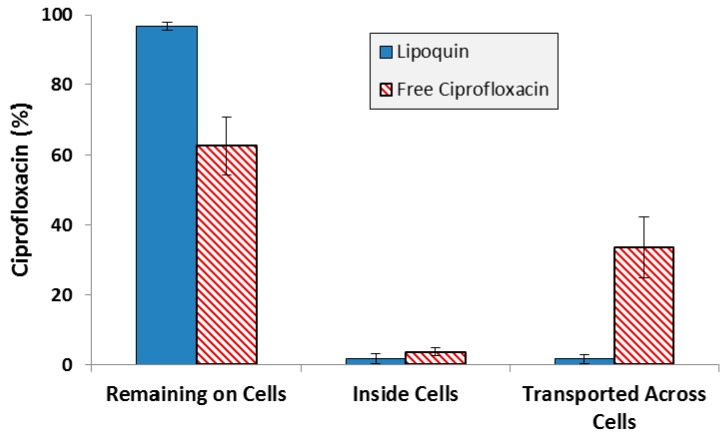
Distribution of ciprofloxacin for Lipoquin and free ciprofloxacin four hours after deposition on Calu-3 cells. Adapted with permission from [38]. Copyright 2012 Springer.

While one of the criteria for selection of the specific liposome formulation used in Lipoquin was its pharmacokinetic release profile in mice [33,36], those studies were conducted following intratracheal administration in mice, not aerosolization. Additional experiments to confirm the retention of the functional properties of Lipoquin during nebulization was conducted by deposition of aerosolized Lipoquin directly on Calu-3 cells inside a transwell insert within a modified glass twin impinger [38]. The Calu-3 sub-bronchial epithelial cell line was chosen as it possesses similar *in vivo* characteristics to native epithelium in terms of morphology, electrical resistance and mucous production. The amount of ciprofloxacin on the cell surface, within the cells, and transported across the cells was quantified over a 4 h period [38]. There was slow uptake of ciprofloxacin into the cells and through the cells, with >95% remaining on the cell surface for Lipoquin, compared to 60% for the free ciprofloxacin control, demonstrating that the liposome integrity was not compromised following aerosolization and deposition on the cell surface (Figure 7). These combined results demonstrate that the essential attributes of the functionality of the liposomes in Lipoquin and Pulmaquin—size, composition and morphology—were effectively chosen and shown to be stable to the nebulization process, essential for an inhaled liposomal aerosol.

### 2.3. In Vitro Characterization and Stability of Liposomal Ciprofloxacin

GMP and GLP batches of Lipoquin have been manufactured at 10, 50 and 100 L scales, characterized through *in vitro* analysis including stability studies through 24 or 36 months at refrigerated conditions, and utilized in the preclinical and clinical development programs of Lipoquin and Pulmaquin. The concentration of ciprofloxacin is unchanged over 24 months’ storage at refrigerated conditions and no ciprofloxacin degradation products have been observed. The liposomes are composed of cholesterol and HSPC [1]. The concentration and purity of cholesterol in the lipid membrane is unchanged when stored for 24 months at refrigerated conditions. Phosphatidylcholine (PC) can undergo acid- or base-catalyzed hydrolysis to form lysoPC and a fatty acid [45]; Lipoquin’s pH was selected to minimize lipid hydrolysis of HSPC.

The physical stability of the liposomes was verified by maintenance of the physical appearance, drug encapsulation, vesicle size distribution and drug release properties during stability studies for at least 24 months under refrigeration. There is no meaningful change in the IVR profile of Lipoquin over 24 months’ storage at 2–8 °C or six months’ storage at room temperature conditions. The drug encapsulation routinely exceeds 98% at release, and there is no decrease in drug encapsulation over 24 months at 2–8 °C; *i.e.*, no leakage of encapsulated drug from intact liposomes or liposome degradation causing complete drug release from some liposomes [42,46].

**Figure 8:**
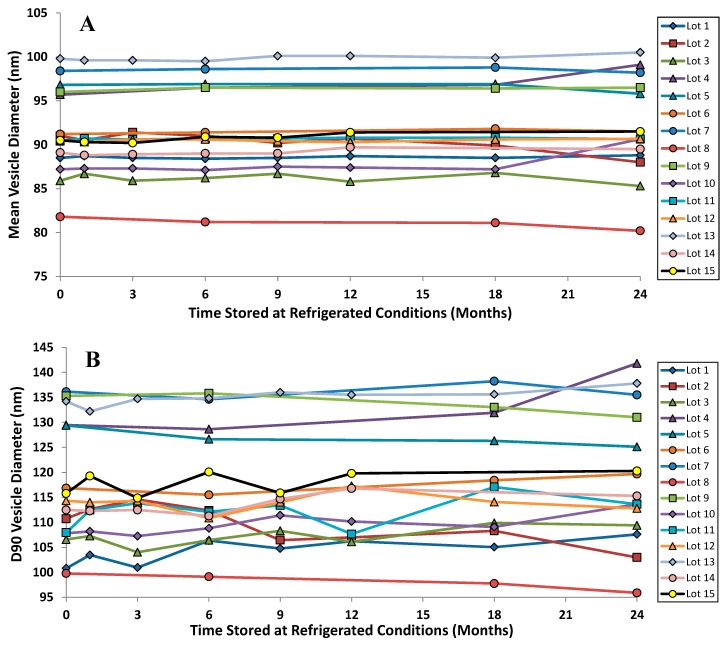
The vesicle size distribution on stability for the first 15 GMP lots of Lipoquin. There was no change in (**A**) the mean vesicle size and (**B**) the D90 size after refrigerated storage for 24 months. Adapted with permission from references [41,42]. Copyright 2013 RDD online.

The vesicle size distribution for the first 22 GMP batches manufactured at three different scales (10, 50 and 100 L) were comparable at release with a mean (± SD) size by dynamic light scattering (DLS) of 91.3 (± 4.3) nm [42,46]. It is typical for vesicle size determinations by indirect measurements like DLS to exceed the physical size estimated by cryo-TEM [38]. DLS measures the hydrodynamic diameter of the vesicles which is influenced by associated ions and surface structure. Cryo-TEM can also be confounded by size-sorting and the preferential exclusion of larger particles from the center of the grid where the film is thin [47]. Regarding stability, there is no change in the vesicle size distribution after up to two years refrigerated storage: the mean vesicle size and the D90 values are shown for the first 15 GMP batches of Lipoquin (Figure 8). Neither the FDA draft liposome guidance [48], nor the ICH Quality Guidelines on Stability (Q1A–Q1F) nor Specifications (Q6A–Q6B) [49] make specific recommendations on the acceptable variation in liposome vesicle size across lots or on stability; however, these vesicle size data demonstrate that the manufacturing process is well controlled and produces liposomes of consistent size.

## 3. Preclinical Development of Liposomal Ciprofloxacin

Extensive preclinical safety studies of inhaled Lipoquin, and Pulmaquin in rats and dogs for variable exposure durations have been conducted to support clinical development of these two products (unpublished data). The efficacy of Lipoquin, Pulmaquin and “Pulmaquin-prototype” formulations in various *in vitro* and *in vivo* models is described below.

### 3.1. Pharmacology

As will be discussed below, Lipoquin and “Pulmaquin-prototype” formulations are both efficacious in a biofilm inhibitory concentration assay against multiple clinical isolates of *P. aeruginosa* (unpublished data) and increased survival in a murine model of *P. aeruginosa* lung infection (unpublished data). Lipoquin also increases survival in murine models of *F. tularensis* [50] and *Y. pestis* [51] lung infection and reduced clinical signs in *C. burnetii* lung infection (a non-fatal infection) [52]. Both Pulmaquin and Lipoquin are efficacious against non-tuberculosis mycobacteria (NTM) *Mycobacterium avium* and *Mycobacterium abscessus* in biofilm and macrophage infection models, as well as in mouse models [26,27,28]. Thus, inhaled Pulmaquin or Lipoquin may be efficacious in patients harboring *P. aeruginosa*, *F. tularensis*, *Y. pestis*, *C. burnetii*, *M. avium* or *M. abscessus* lung infections.

### 3.2. Efficacy against Pseudomonas aeruginosa: In Vitro Studies

The efficacy of Lipoquin, free ciprofloxacin (FCI) and “Pulmaquin-prototype” formulations were tested against biofilms of *P. aeruginosa* from three genotypically distinct clinical isolates [12]. The “Pulmaquin-prototype” formulation tested in this study was a 1:1 volume-to-volume (*v*/*v*) mixture of Lipoquin (50 mg/mL) and FCI, 30 mg/mL. Lipoquin, “Pulmaquin-prototype”, and FCI have similar inhibitory concentrations against biofilms of *P. aeruginosa*.

**Figure 9:**
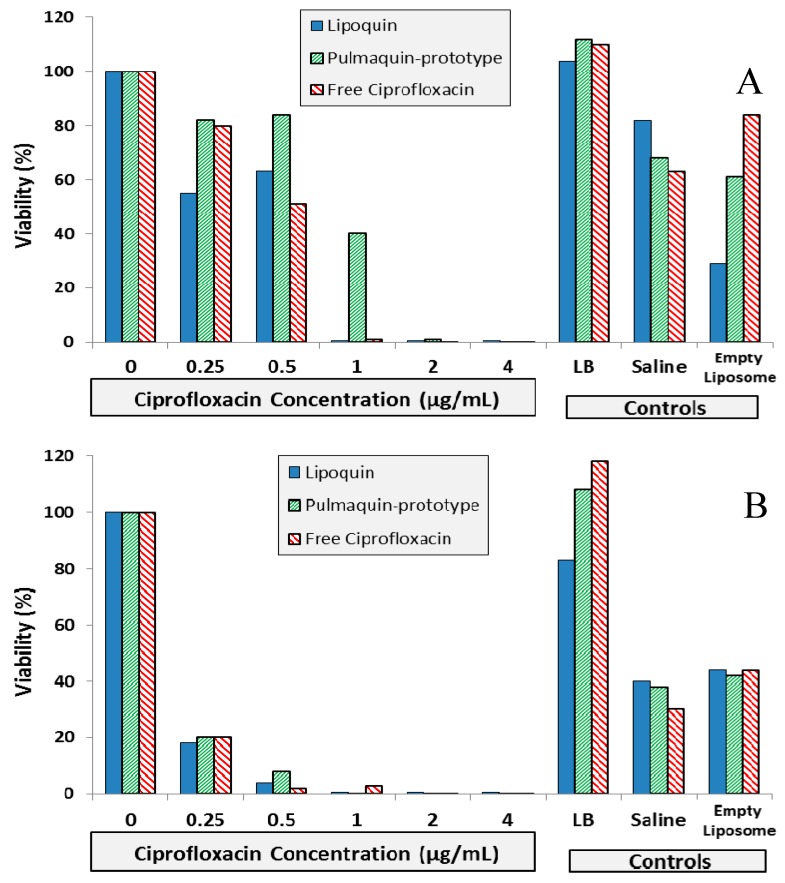

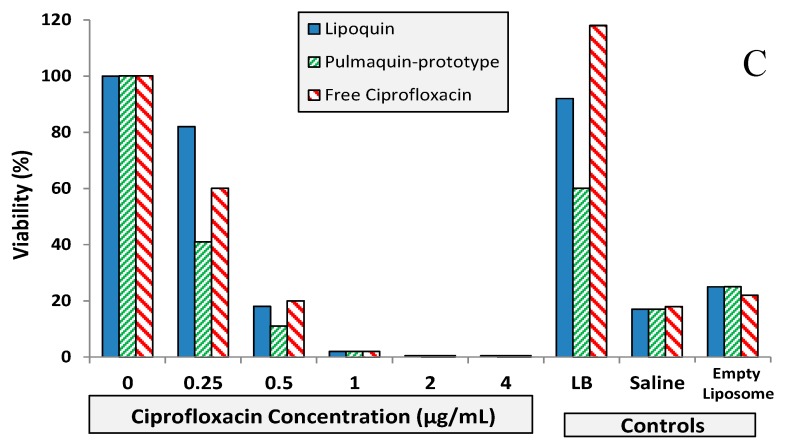
BIC assay results for Lipoquin, Pulmaquin-prototype and free ciprofloxacin formulations in (**A**) CF sputum isolate (Strain #26), (**B**) ventilator-associated pneumonia endotracheal aspirate isolate (Strain #19), and (**C**) CF sputum isolate (Strain #57a). Controls include LB cell medium, saline and empty liposomes. Results show the mean viability of biofilm of two separate experiments based on OD readings made in triplicate for each experiment. The mean OD value for each formulation was compared to that of the LB medium control to determine the percent reduction in biofilm biomass (viability).

The biofilm inhibitory concentration (BIC) assay results for the isolates are shown in Figure 9. For Strain #26 (CF sputum isolate), Lipoquin and free ciprofloxacin had a 99% reduction in viability at a concentration of 1.0 µg/mL; whereas the “Pulmaquin-prototype” formulation needed a concentration of 2.0 µg/mL to obtain a 99% reduction (Figure 9A). For Strain #19 (ventilator assisted pneumonia isolate), the Lipoquin and “Pulmaquin-prototype” formulations both had a 99% reduction at 1.0 µg/mL, with the free ciprofloxacin having a comparable reduction at 2.0 µg/mL (Figure 9B). The results of the third isolate (Strain #57a from CF sputum), mimicked those of the other two strains (Figure 9C). Therefore, Lipoquin, “Pulmaquin-prototype” and free ciprofloxacin had comparable efficacy inducing a 99% reduction in viability of *P. aeruginosa* at concentrations of 1.0–2.0 µg/mL. Since the biofilm assay has no clearance pathway, in contrast to the *in vivo* dynamic of rapid systemic absorption of free ciprofloxacin, the free drug remains in contact with the biofilm and continues to subject it to its antimicrobial properties potentially inflating the efficacy of the free ciprofloxacin arm.

### 3.3. Efficacy against Pseudomonas aeruginosa: In Vivo Studies

Both Lipoquin and “Pulmaquin-prototype” formulations increased survival (Tables A2 to A4 in the Supplementary Information) in a well-characterized model of clinical exacerbation in CFTR-deficient mice with PA lung infection [53]. Outcome measures included survival and morbidity, as assessed by change in initial body weight and clinical signs, which were monitored one- to two-times daily. A 6-point scale (0–5) for scoring clinical signs is provided in Supplementary Information (Table S1) with a score of 0 indicating normal activity and healthy appearance and a score of 5 indicating moribund health or death.

Administration of Lipoquin or “Pulmaquin-prototype” formulations resulted in higher survival and clinical scores compared to TOBI or the negative control (diluent) in treating PA-infected mice bearing mutations in *Cftr*. However, given the relatively small sample sizes per group for the outcome measures chosen, the results were not statistically significant due to the large study-to-study variability. Treatment was not associated with an adverse effect, and in most experiments, mice treated with either Lipoquin or “Pulmaquin-prototype” formulations had higher survival rates and better clinical scores compared to mice treated with diluent. Therefore, this study supports the hypothesis that once daily treatment with Lipoquin or Pulmaquin inhaled directly to the lungs may be efficacious in patients with CF or NCFB.

### 3.4. Efficacy against Biodefense Pathogens

Biological weapons can be developed from inhaled pathogens that are intracellular, which target and infect lung cells including macrophages. Examples are *Francisella tularensis, Yersinia pestis*, *Coxiella burnetii*, which when inhaled can cause pneumonic tularemia, pneumonic plague, and Q fever, respectively.

Once inhaled, there is a window of time (usually 24 h or longer, depending on the pathogen) before clinical symptoms of these infections are seen; delivering antibiotics in this time window is called “post-exposure prophylaxis (PEP)”. Delivering antibiotics after symptoms are evident is called “treatment”. It is clearly desirable to administer antibiotics as quickly as possible before symptoms develop; therefore, studies testing efficacy of antibiotics for PEP are important.

The antibiotics for these infections are conventionally given orally or intravenously. However, delivering these antibiotics encapsulated within liposomes offers at least two advantages over free (unencapsulated) drug given orally or IV. First, the infected phagocytic cells, such as macrophages and monocytes, that have ingested the pathogens, also ingest the liposomes, which brings the antibiotic and pathogens into close proximity intracellularly, thereby greatly increasing the antibiotic’s bioavailability. Since infected macrophages are also the means to spread the infection within the host, the liposomal formulation would have superior efficacy, especially in the early phases of the lung infection. Second, the slow release of the antibiotic from the liposome maintains high sustained concentrations in the lungs.

The efficacy of Lipoquin for PEP against pneumonic tularemia [50] and Q fever was established in rodent models [51]. Lipoquin has also shown efficacy for PEP against pneumonic plague in a preliminary study [52].

#### 3.4.1. Pneumonic (Respiratory/Inhalational) Tularemia—Lipoquin Efficacy against *F. tularensis*

*F. tularensis* can infect animals and humans causing tularemia. Rabbits and rodents are especially vulnerable. The most severe form of this disease occurs when the pathogen is inhaled causing pneumonic tularemia, and only a low dose of bacteria is needed. Symptoms include cough, chest pain, and difficulty breathing, but if untreated has a fatality rate of up to 30% [54]. Pneumonic tularemia is typically treated with oral ciprofloxacin or doxycycline for 14 days, which can reduce the fatality rate to about 2% [54]. However, high levels of relapse have been seen in patients treated with oral doxycycline [55] or ciprofloxacin [56] following a tularemia outbreak in Spain, which was likely caused by a less virulent strain [57]. A more virulent strain of *F. tularensis*, such as Schu S4, would likely be developed as a biological agent; however, there are no human data for such an exposure, and animal studies of pneumonic tularemia from Schu S4 have found oral ciprofloxacin for 14 days provided no protection with 100% mortality [58]. Therefore, more efficacious approaches for PEP for pneumonic tularemia are needed.

**Figure 10:**
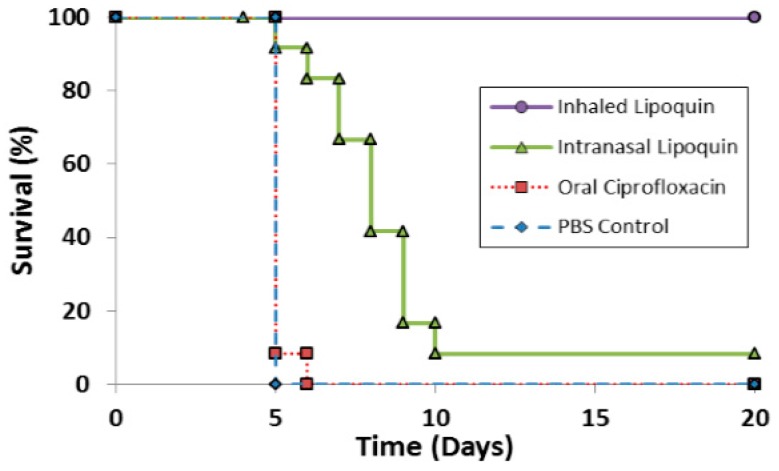
Therapeutic efficacies of oral ciprofloxacin, intranasal Lipoquin, and inhaled Lipoquin aerosol against inhalational *F. tularensis* Schu S4 infection in mice. Groups of 12 BALB/c mice were challenged with *F. tularensis* Schu S4 via the aerosol route and treated at 24 h post-challenge with 50 mg/kg of oral ciprofloxacin (red squares, dotted line), 50 mg/kg of intranasal Lipoquin (green triangles, solid line), a 1-mg/kg lung dose of aerosolized Lipoquin (purple circles, solid line), or intranasal phosphate buffered saline (PBS) (blue diamonds, dashed line). Graph shows the survival of mice treated with a single dose of antibiotic. Reprinted with permission from [50]. Copyright 2014 American Society for Microbiology.

The efficacy of prototype formulations of liposomal ciprofloxacin against *F. tularensis* has been tested in several studies [59,60] which have been reviewed [61]. Recent efficacy studies with Lipoquin for PEP in BALB/c mice have been reported [50]. A single dose of aerosolized Lipoquin provided 100% protection and significantly increased survival (*p* < 0.005) compared to untreated controls, oral ciprofloxacin, and intranasal Lipoquin (Figure 10). In contrast, a single dose of intranasal Lipoquin provided only 10% protection and a single dose of oral ciprofloxacin provided no protection. The inferior efficacy of intranasal Lipoquin relative to aerosolized Lipoquin may be related to the mismatch in the location of the infectious agent - which is deposited uniformly throughout the lung - versus intranasal instillation of the therapeutic which may be localized only to a small region of the lung [62]. The efficacy of three and five doses of intranasal Lipoquin *versus* oral ciprofloxacin is shown in Figures S1A,B in the Supplementary Information.

PK studies showed that aerosolized Lipoquin (1 mg/kg) provides an AUC_0–24_ in the lungs that is over 80-fold greater than oral ciprofloxacin (50 mg/kg), which is rapidly eliminated from the lungs (half-life 4.2 h) whereas aerosolized Lipoquin has an 80-percent longer half-life (7.4 h) [50].

In conclusion, Lipoquin has demonstrated very high efficacy against both a virulent strain Schu S4 and a less virulent strain LVS of *F. tularemia*, with a single dose providing 100% protection [50]. Therefore, Lipoquin holds promise to shorten the prophylactic regimen, which is currently 14 days for oral ciprofloxacin or doxycycline [54]. Lipoquin, perhaps when combined with oral or IV ciprofloxacin, may also be efficacious against systemic tularemia, which can develop from the pneumonic form. Further studies are needed to test this hypothesis as well as in a non-human primate model, which is validated to represent the disease in humans, that is needed for approval for the treatment and prophylaxis of *F. tularemia* infection under the FDA “Animal Rule” [63].

#### 3.4.2. Q Fever–Lipoquin Efficacy against *C. burnetii*

*Coxiella burnetii* causes Q fever, which leads to acute severe flu-like symptoms that typically appear in 2–3 weeks but then resolve in about 2 weeks; it is usually non-fatal with a very low mortality rate (<2%) [64,65]. The alveolar macrophage is the target cell in the lungs when *C. burnetii* aerosol is inhaled [64]. Chronic Q fever occurs in less than 5% of acutely infected patients, with the greatest risk being pregnant women, immunosuppressed patients, those with pre-existing heart defects; however, 60%–70% of these patients develop endocarditis, which is fatal in 25%–60% in untreated patients [64,65,66]. The 10-year mortality rate with chronic treatment is 19% [67].

The bacteria is zoonotic typically residing in goats, cattle, sheep, *etc.* that spread it to humans [64]. However, it is also a potential biological agent since it is very infectious with a single bacterium able to cause disease in susceptible people; therefore, the Centers for Disease Control (CDC) classifies it as a Category B agent [68].

The recommended treatment for acute Q fever is 100 mg oral doxycycline, every 12 h for 2–3 weeks; for chronic Q fever it is 100 mg oral doxycycline every 12 h and 200 mg hydroxychloroquine every 8 h for 18 months up to 4 years for endocarditis or vascular infection [67,69]. The hydroxychloroquine acts as a lysosomotropic alkalinization agent since the *C. burnetii* resides intracellularly in phagolysosomes that are acidic, pH 4.8, and since the activity of doxycycline is diminished by low pH, the alkalinization agent raises the pH of the phagolysosome to 5.7, which is optimum for doxycycline [70]. However, long-term treatment with hydroxychloroquine may cause retinal toxicity in children; therefore, alternative long-term treatments are a fluoroquinolone (e.g., moxifloxacin or levofloxacin) with rifampin or trimethoprim/sulfamethoxazole with doxycycline [67].

Of note for both acute and chronic treatment with doxycycline is the evidence of resistance; *i.e.*, in a patient who had chronic Q fever endocarditis and died following unsuccessful antibiotic treatment, at least one doxycycline-resistant strain of *C. burnetii* was isolated [71]. Thus, there is a need to develop new therapies that are not limited by resistance to doxycycline and improve treatment, perhaps shortening it for both acute and chronic Q fever. Ciprofloxacin is a viable therapy since the MIC for *C. burnetii* is 4 µg/mL for the acute infection model isolate (Nine Mile) and 7 of 8 clinical isolates from Greece; for the chronic infection model isolate Q212 and one clinical Greek isolate the MIC was 8 µg/mL [72]. It is expected that the liposomal formulation will help achieve bacteriostatic levels in the cell. There are also limited reports of oral ciprofloxacin being efficacious for treating chronic Q fever endocarditis, but not usually acute Q fever; as mentioned above, ciprofloxacin combined with rifampin is an alternative therapy for chronic Q fever in children [67].

**Figure 11:**
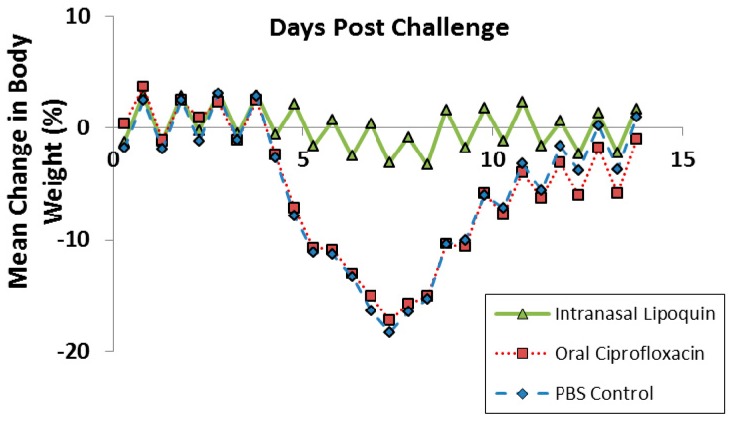
Efficacy of oral ciprofloxacin or intranasal Lipoquin against *C. burnetii*. Mean percentage change in body weight of mice (*n* = 10) infected with *C. burnetii* and treated for 7 days post-challenge. Reprinted with permission from [52]. Copyright 2014 American Society for Microbiology.

Lipoquin was evaluated as a post exposure therapeutic for *Coxiella burnetii* acute infection [52]. Male A/Jola (A/J) mice (*n* = 10/group) were challenged with aerosolized *C. burnetii* (2.8 × 10^6^ genome equivalents) and then treated starting 24 h later for seven consecutive days with either intranasal Lipoquin (50 mg/kg) once daily, oral (free) ciprofloxacin (50 mg/kg) twice daily, or oral phosphate-buffered saline (PBS) control twice daily and then monitored for 14 days post-challenge. Mice treated with oral ciprofloxacin or PBS on Day 7 had a decrease in body weight, which on Day 7 was 20%, and on Days 4–9 exhibited piloerection, arched backs, and dehydration (Figure 11). Conversely, mice treated with intranasal Lipoquin had no weight loss (*p* < 0.001 from day 4) or clinical signs. *C. burnetii* infection significantly increases the weight of lungs and spleen [52]. Mice treated with intranasal Lipoquin had less significantly attenuated weight increases in these organs compared to oral ciprofloxacin (*p* < 0.05 lungs, *p* < 0.001 spleen) [52]. Similarly, in the Lipoquin treated mice, the bacterial CFUs in the lungs and spleen were 10–100-fold lower than those treated with PBS, with the decrease in the lungs being significant (*p* < 0.001) *versus* oral ciprofloxacin [52]*.*

**Figure 12:**
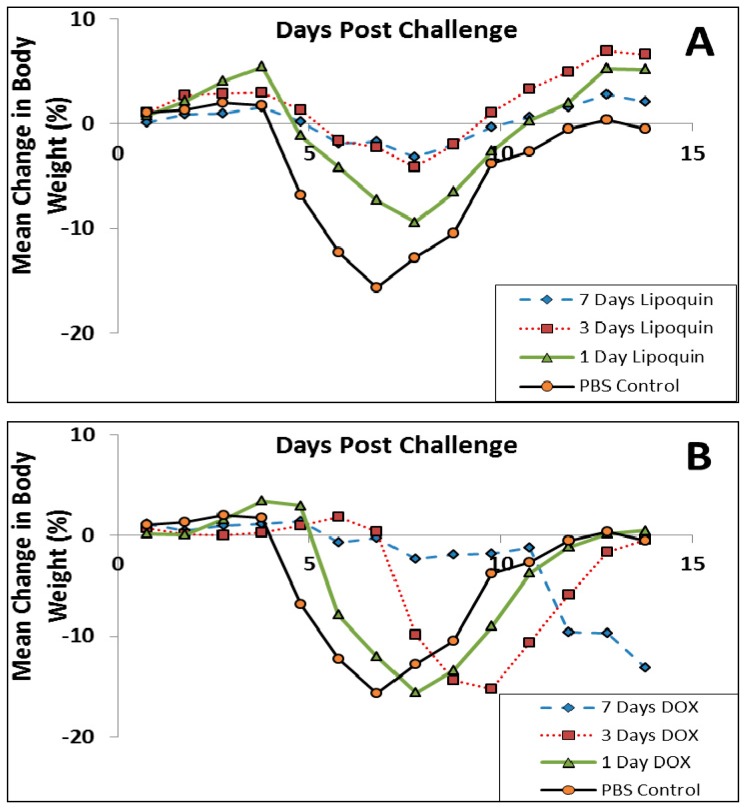
Efficacy of (**A**) intranasal Lipoquin or (**B**) oral doxycycline (DOX) against *C. burnetii versus* PBS-treated control mice. Mean percentage change in body weight of mice (*n* = 10) infected with *C. burnetii* and treated for 1 day, 3 days, or 7 days post-challenge. Reprinted with permission from [52]. Copyright 2014 American Society for Microbiology.

Since Q fever is typically treated with oral doxycycline [67], the study was repeated comparing intranasal Lipoquin (50 mg/kg) once daily, oral doxycycline (50 mg/kg) twice daily, or oral PBS twice daily with dosing occurring for 1, 3 and 7 days followed by monitoring for 7 and 14 days (*n* = 10 mice per group per time point) [52]. By Day 7, PBS-treated mice had a 15% decrease in body weight (Figure 12), and 80% had clinical signs, including piloerection, at least once during the 7 days [52]. Doxycycline-treated mice had a 13%–15% decrease in body weight, but the onset was delayed compared to the PBS-treated mice (Figure 12B). The extent of weight loss correlated with clinical signs that were observed in 70% of the mice in all doxycycline-treatment groups. In contrast, for the mice treated with Lipoquin, the extent of the weight loss starting from Day 8 was significantly less than the comparable doxycycline-treated groups (*p* < 0.001) (Figure 12A). The mice treated with a single dose of Lipoquin also had significantly less weight loss on Days 12 to 14 than the doxycycline mice treated for 1, 3 or 7 days (*p* < 0.01). Clinical signs were seen in 50% of mice dosed for 1 day with Lipoquin and 0% in mice dosed for 7 days.

Comparing the changes in lung and spleen weights and bacterial colonization at day 14 for mice treated for 7 days, the mice treated with Lipoquin had significantly smaller increase in lung weight compared to doxycycline (*p* < 0.001) and less increase in spleen weight compared to PBS (*p* < 0.05) but not the doxycycline treatment [52]. The Lipoquin-treated mice also had significantly lower CFU in the lungs and spleen compared to the doxycycline-treated mice (*p* < 0.001) [52].

In summary, when compared to mice treated with 7 days of oral ciprofloxacin, 7 days of intranasal Lipoquin protected mice from clinical signs and significant loss of body weight. The Lipoquin mice also had significantly smaller increases in lung and spleen weight and bacterial count in the lungs. The increases in lung and spleen weight are typical findings due to infection; both organs are relevant as the lung is the site of infection and the spleen indicates the magnitude to which is infection is disseminating systemically. Similarly, when compared to mice treated with 7 days of oral doxycycline, after 7 days of intranasal Lipoquin, the mice had significantly less increase in lung weight and lower bacterial counts in the lungs and spleen. Remarkably, one dose of intranasal Lipoquin also resulted in significantly less body weight loss than up to 7 days of oral doxycycline. Thus, Lipoquin is a promising therapy for Q fever that may offer improved therapeutic outcomes *versus* oral doxycycline, the current recommended therapy.

## 4. Clinical Development of Liposomal Ciprofloxacin

The initial clinical development of inhaled liposomal ciprofloxacin was a Phase 1 trial in 20 healthy volunteers using Lipoquin [9]. This was a safety, tolerability and pharmacokinetic study that included single dose escalation (3–9 mL doses loaded in the nebulizer, equivalent to 150–450 mg of ciprofloxacin expressed as the hydrochloride salt), followed by dosing in a cohort of subjects for one week with 6 mL Lipoquin once each day [9]. Administration of the liposomal formulation by inhalation was well tolerated and no serious adverse reactions were reported [9]. The pharmacokinetic profile obtained by measurement of blood levels of ciprofloxacin following the inhalation of the liposomal formulation was consistent with the profile from sustained release of ciprofloxacin from liposomes, supporting once daily dosing; the blood levels of ciprofloxacin were much lower than those that would be observed following administration of therapeutic doses of ciprofloxacin by injection or via the gastrointestinal tract. These initial clinical data thus validated the preclinical design of a once-daily inhaled product.

**Figure 13:**
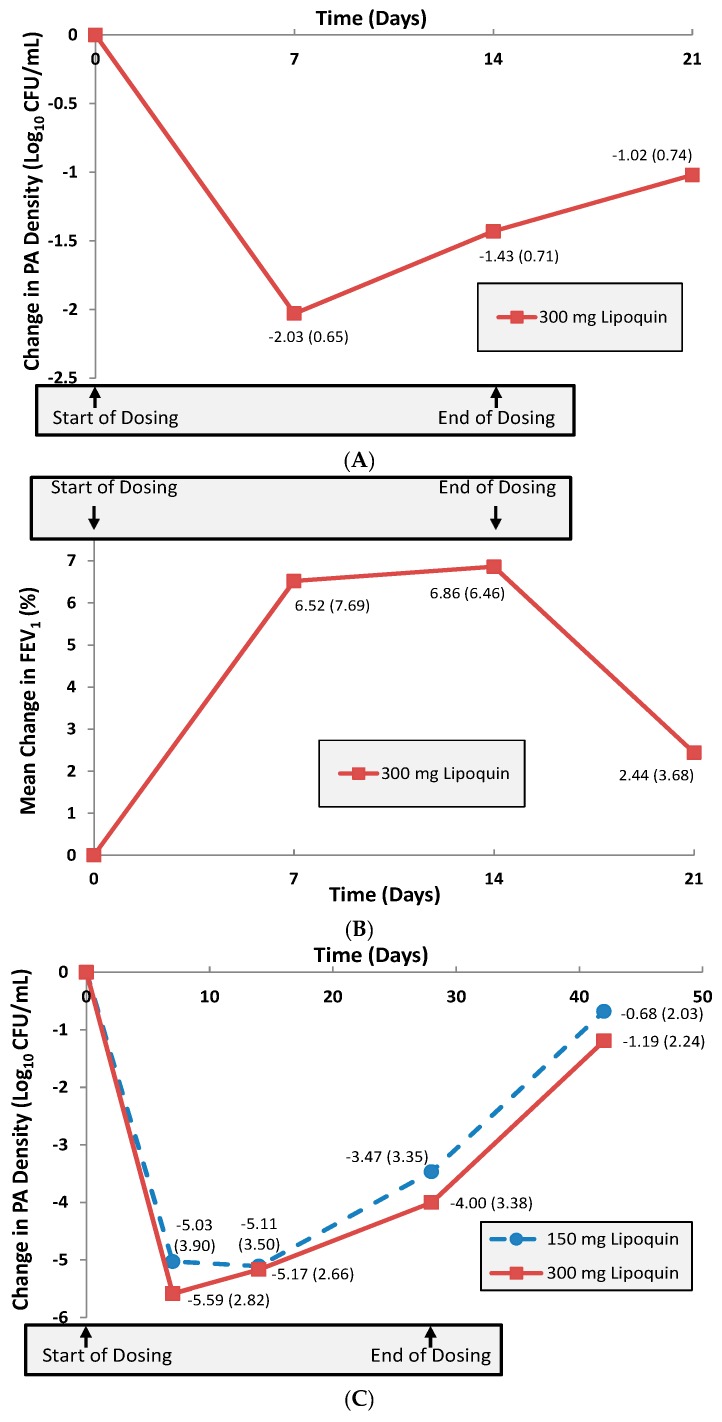
(**A**) Mean change (±SD) in sputum PA Log_10_ CFU from baseline (*n* = 21 CF Patients). Adapted with permission from [9]. (**B**) Mean change (±SD) in % FEV_1_ from baseline (*n* = 21 CF Patients). Adapted with permission from [9]. (**C**) Mean change (±SD) in sputum PA Log_10_ CFU from baseline (*n* = 36 NCFB Patients). Adapted with permission from [9]. Copyright 2010 RDD online.

A multi-center 14-day Phase 2a trial was then conducted in 21 adult CF patients to investigate initial safety, efficacy and pharmacokinetics of once-daily inhaled liposomal ciprofloxacin (Lipoquin) in this population [9]. The primary efficacy endpoint in this Phase 2a study was the change from baseline in the sputum *Pseudomonas aeruginosa* (PA) colony forming units (CFU), an objective measure of the reduction in pulmonary bacterial load of this pathogenic organism which is associated with morbidity in these patients [1]. The study demonstrated that the CFUs decreased by a mean 1.43 log over the 14-day treatment period (*p* < 0.0001) (Figure 13A) [9]. Evaluation one week after study treatment was discontinued showed that the PA bacterial density in the lung was still reduced from baseline levels without additional antibiotic use [9]. Pulmonary function testing as measured by the forced expiratory volume in one second (FEV_1_) showed a significant mean increase of 6.8% from baseline after 14 days of treatment (*p* = 0.04) (Figure 13B) [9]. The study drug was well tolerated, and there were no serious adverse events reported during the trial [9].

It was the remarkable tolerability in CF that encouraged the exploration of Lipoquin in non-cystic fibrosis bronchiectasis (NCFB) patients colonized with PA; this population of patients historically failed to show a positive clinical response to inhaled antibiotics, in contrast to CF patients [73].

The first trial in NCFB was an open-label, four-week, multi-center study of efficacy, safety and tolerability of once-daily 6 mL of Lipoquin in 36 adult patients [9]. The patients were randomized into two equal size groups, one receiving 3 mL and the other receiving 6 mL of inhaled liposomal ciprofloxacin. The primary efficacy endpoint was the change from baseline in the sputum PA CFUs. The 3 and 6 mL doses of inhaled liposomal ciprofloxacin in the evaluable patient population demonstrated similar significant mean decreases against baseline in the PA CFUs over the 28-day treatment period of 3.5 log (*p* < 0.001) and 4.0 log (*p* < 0.001) units, respectively (Figure 13C) [9]. This result suggested that the 3 mL dose was likely already at the top of the dose-response curve for anti-pseudomonal activity. With regard to safety, there were no statistically significant changes in lung function for the evaluable patient population at the end of treatment as measured by the normalized forced expiratory volume in one second (FEV_1_, % predicted); in NCFB, in contrast to CF, there appears to be no reversible component of FEV_1_ that improves in response to treatment with inhaled antibiotics, and FEV_1_ is therefore measured for safety rather than efficacy. Lipoquin was well tolerated: no bronchodilator use was mandated or needed before administration of the study drug. In the 3 mL group, respiratory drug-related adverse reactions were only mild. Three serious adverse events were observed in each dose group, with only one of the six classified as possibly drug-related in the 6 mL group. This particular patient suffered from a viral infection (shingles) early in the treatment period that might have been a confounding factor leading ultimately to a respiratory exacerbation requiring hospitalization.

While the data with Lipoquin were encouraging, the combination of Lipoquin and unencapsulated ciprofloxacin were evaluated to determine if additional clinical benefits might be gained from a more rapid peak ciprofloxacin concentration. Concentrated solutions of ciprofloxacin ranging from 20 to 30 mg/mL (as a hydrochloride salt) were developed to be mixed in a 1:1 *v*/*v* ratio with the liposomal ciprofloxacin formulation (Lipoquin). The rationale for these compositions were to preserve the key desirable attributes of the liposomal formulation (once-daily dosing, good tolerability and safety, ability to penetrate biofilms and action against intracellular infections) with additional potential benefits resulting from the immediately-available component. This product concept was initially explored in preclinical models as described above. In a non-specific lung inflammation model (Aradigm unpublished data), this combination product exhibited potent anti-inflammatory activity which was not anticipated. Independently, scientists from the Virginia Commonwealth University reported findings about the anti-inflammatory effects of liposomal ciprofloxacin (Lipoquin) in human bronchial lung cells stimulated to produce inflammatory cytokines by the lipopolysaccharide (LPS) produced by *Pseudomonas aeruginosa*. LPS produced by this organism is a key virulence-causing factor associated with the respiratory infections due to this microorganism [74].

Further development was thus explored for both the liposomal formulation alone (Lipoquin) as well as the mixture of 3 mL of Lipoquin with 3 mL of free ciprofloxacin (which was later named Pulmaquin). A Phase 1 study in healthy volunteers and in NCFB subjects was conducted with an “Pulmaquin-prototype” formulation and Lipoquin [46,75,76]. Both formulations were safe and well-tolerated in healthy subjects and patients with NCFB. The pharmacokinetic profile of Lipoquin in healthy subjects possessed a long plasma half-life of ~10 h (suggesting a long residence time in the lung, as oral and IV ciprofloxacin have a much shorter elimination half-life), compatible with a single daily treatment regimen [9]. The plasma pharmacokinetic profiles of the “Pulmaquin-prototype” formulation in both healthy subjects and NCFB patients also demonstrated a lung-release limited plasma half-life of ~10 h [46]. However, “Pulmaquin-prototype” resulted in significantly higher plasma *C*_max_ values compared to Lipoquin as would be expected by the presence of the unencapsulated component [46]. The areas under the plasma curve (AUC) and plasma Cmax were more than an order of magnitude smaller following administration of “Pulmaquin-prototype” or Lipoquin, compared to plasma levels of approved doses of oral or IV ciprofloxacin, suggesting that the potential for systemic side-effects, even upon chronic dosing, was significantly reduced [46]. The previously tested anti-pseudomonal activity of 3 and 6 mL of Lipoquin in NCFB patients provided assurance that the 6 mL dose of Pulmaquin would also be maximally effective. An even lower dose of Lipoquin (2 mL) was evaluated to ascertain if the 3 mL dose was at the beginning of the plateau of the dose-response curve, thus risking that some patients may not fully benefit from the antipseudomonal activity of the treatment. These subsequent series of trials were named “ORBIT” (Once-daily Respiratory Bronchiectasis Inhalation Treatment).

The Phase 2b trial with Lipoquin, ORBIT-1, was an international, double-blind, placebo-controlled study in which 95 adult NCFB patients with respiratory infections with PA were randomized. The study design called for four weeks of once-daily inhaled doses of Lipoquin or once-daily inhaled placebo. Two doses of the active drug were included in the study: 100 mg (2 mL) or 150 mg (3 mL) ciprofloxacin, together with their respective placebos. The primary endpoint—the mean change in PA CFUs from baseline to day 28—was met in the full analysis population that included all patients who were randomized, received at least one dose and provided samples for at least two time points. There was a significant mean reduction (*p* < 0.001) of 2.94 log CFUs in the 3 mL Lipoquin group and a significant mean reduction (*p* < 0.001) of 3.84 log CFUs in the 2 mL Lipoquin group compared to their respective placebos. The pooled placebo groups had a mean reduction of 0.44 log CFUs. There was no statistically significant difference between the 2 and 3 mL Lipoquin doses. Lipoquin was well-tolerated and no bronchodilator treatment was mandated before inhaled study treatments. There were no statistically significant differences between the active and placebo groups in the number of patients experiencing at least one respiratory treatment-emergent adverse event. The incidence of serious adverse events (SAEs) was low; there were a total of 6 SAEs and none of them were treatment related. ORBIT-1 therefore confirmed that 3 mL of Lipoquin is a maximally effective liposomal ciprofloxacin dose in the NCFB patients with respect to reduction of the bacterial load of PA in the sputum.

**Figure 14:**
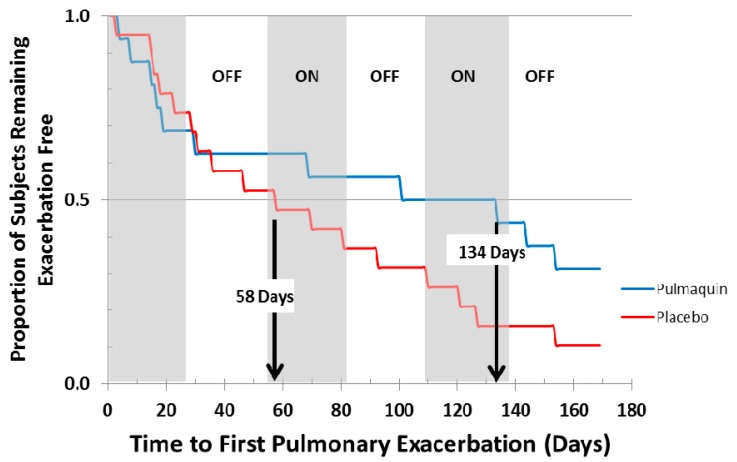
Kaplan–Meier curve showing the time to first pulmonary exacerbation for Pulmaquin and Placebo groups over the 3 treatment cycles (28 days on followed by 28 days off) in the modified intention to treat (mITT) population. Reprinted with permission from [21]. Copyright 2013 BMJ.

ORBIT-2 was a 168 day, multi-center, international, Phase 2b clinical trial with 6 mL Pulmaquin (210 mg) in 42 adult NCFB patients with respiratory infections with PA [21]. In this randomized, double-blind, placebo-controlled trial the patients were treated once-a-day for 28 days with either the active drug, or placebo, followed by a 28 day off-treatment period. This 28-day on-off sequence was repeated three times. Statistical significance was achieved in the primary endpoint—the mean change in PA density in sputum from baseline to day 28 [21]. In the full analysis population (all patients who were randomized, received at least one dose and provided samples for at least two time points), there was a significant mean reduction of 4.2 log PA CFU units in the Pulmaquin group, reflecting an almost sixteen-thousand fold decrease in PA bacterial load, *versus* a very small mean decrease of 0.1 log units in the placebo group (*p* = 0.004) [20]. Secondary endpoint analysis showed that 17 subjects in the placebo group required supplemental antibiotics for respiratory-related infections *versus* 8 subjects in the Pulmaquin group (*p* = 0.05) [21]. The Kaplan–Meier analysis showed that the median time to first pulmonary exacerbation in the per protocol population evaluation increased from 58 days in the placebo group to 134 days in the active treatment group and was statistically significant (*p* < 0.05, log rank test) (Figure 14) [21].

**Figure 15:**
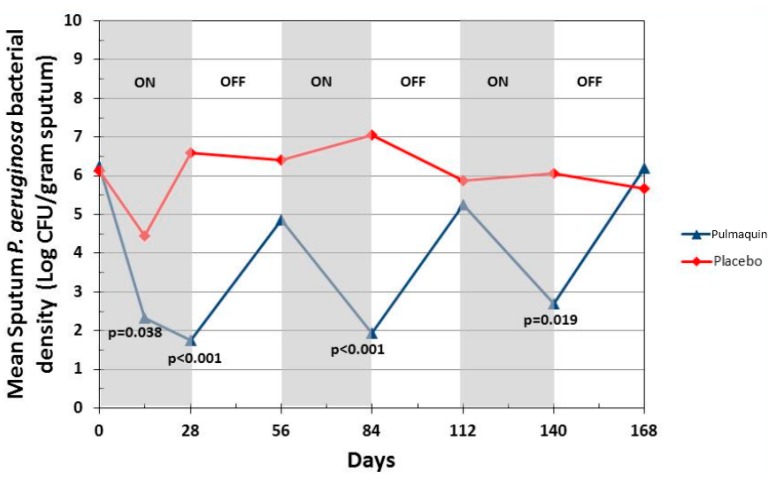
Change in mean sputum PA bacterial density across the 3 treatment cycles (28 days on followed by 28 days off) for Pulmaquin and Placebo groups in the modified intention to treat (mITT) population. Reprinted with permission from [21]. Copyright 2013 BMJ.

Pulmaquin was well tolerated and there were no significant decreases in lung function, as measured by FEV_1_ at 28 days in either group [21]. Overall, the incidence and severity of adverse events were similar in both the placebo and treatment groups; however, Pulmaquin had a superior pulmonary safety profile reflected in the number and severity of pulmonary adverse events [21]. Further statistical analysis concluded that the reduction from baseline in PA CFUs with Pulmaquin was rapid and persistent throughout the treatment cycles as exemplified by the statistically significant reductions of the mean log CFU values in the Pulmaquin group *versus* placebo at day 14 and day 28 during the first treatment cycle, as well as at the end of the second and third cycles of treatment (days 84 and 140, respectively) (Figure 15).

Based on the totality of the preclinical and clinical evidence, 6 mL of once-daily Pulmaquin was selected for the Phase 3 program in NCFB. The ORBIT-3 and ORBIT-4 trials are prospectively-designed, randomized, placebo-controlled, international, multi-center studies in approximately 255 patients per trial. The patients must be colonized with PA. The trials consist of 6 double-blind cycles of 28 days on/28 days off treatment, followed by a 28 day, open-label extension with all patients receiving Pulmaquin. The primary endpoint is the time to first pulmonary exacerbation; other endpoints include the number of pulmonary exacerbations, the number of severe pulmonary exacerbations as well as QoL. Lung function tests are conducted throughout the study for safety monitoring. A battery of microbiology testing is also conducted to investigate antibacterial activity as well as any trends of emergence of resistance. These trials were fully enrolled in September 2015 [77].

## 5. Next Generation Formulations of Liposomal Ciprofloxacin

There are a number of patient populations with lung infections that may optimally be treated by different antibiotic release profiles, depending upon the location of the infection (e.g., extracellular *versus* intracellular [37]), the presence of biofilm, and the sensitivity of the infection to the antibiotic. Specifically for intracellular infections, e.g., macrophages harboring NTM [37], it may be more effective to retain the majority of the drug in the liposomes until after the liposomes are phagocytosed by the macrophages, thus necessitating a delayed or slow release formulation. In contrast, for patients colonized with a bacterial strain with a higher MIC, treatment may be more effective if there is a high initial bolus of drug, or a faster drug release profile, or a combination of the two, to more effectively kill the pathogens with high MICs in the lung.

It would be highly desirable if the drug release rate could be modulated using a “parent formulation” requiring minimal or no changes in its composition to personalize therapy to suit an individual patient’s needs. While there are many inhalation devices on the market including DPIs, MDIs, nebulizers and soft-mist inhalers which can be selected to meet the specific needs of an individual patient [78], there are limited examples of formulations or drugs that can be personalized to meet the therapeutic requirements of a specific patient. However, this paradigm may soon change as it has recently become possible to modify the drug release profile of a liposome formulation through simple means to produce a faster releasing formulation [79] or a slower releasing formulation [80].

**Figure 16:**
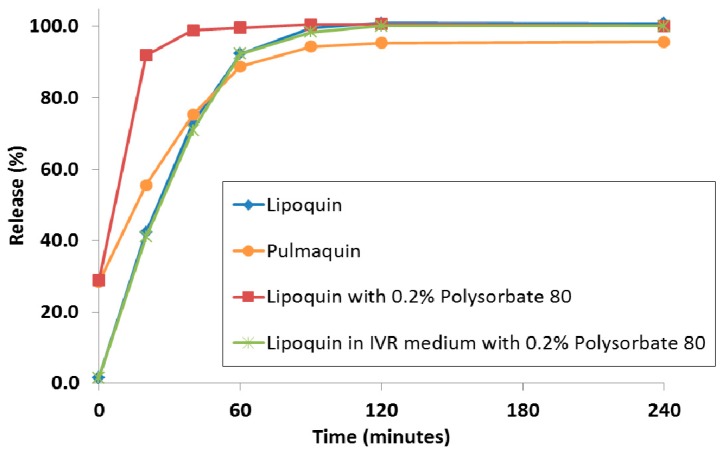
The *in vitro* release profiles of Lipoquin, Pulmaquin, Lipoquin with 0.2% polysorbate 20, and Lipoquin in IVR medium containing 0.2% polysorbate 20. Duplicate samples were analyzed at each time point. The IVR assay is described in [43]. Reprinted with permission from [79].

Historically, to achieve a faster releasing liposome formulation, the composition of the liposome was modified using different lipids or by reducing the proportion of cholesterol in the lipid membrane. There was no way to achieve a faster drug release profile without modification of the liposome composition and this new liposome composition would require a separate manufacturing campaign. It was recently shown that the simple addition of surfactant; e.g., polysorbate 20 or polysorbate 80, to the liposomes in a hypotonic environment resulted in faster drug release in an IVR assay [79]. Traditionally, interactions of surfactant with liposomes were investigated to model the solubilization and break-down of biological membranes. In contrast, in these studies, the hypotonic environment, and the resulting osmotic swelling of the liposomes, allowed for the surfactant to increase the permeability of the liposomes [79]. Figure 16 shows the distinct release profiles for Lipoquin, Pulmaquin, and Lipoquin diluted with 0.2% polysorbate 80. As a negative control, the addition of the same concentration of surfactant into the IVR medium (0.2% polysorbate 80) had no effect on drug release from Lipoquin confirming that the faster release was not simply a consequence of the presence of surfactant in the release medium (Figure 19).

One surfactant-modified liposome formulation (Lipoquin containing 0.4% polysorbate 20) was stable for two years at refrigerated conditions with no meaningful change in visual appearance, pH, vesicle size or drug encapsulation [81]. The formulation was stable to mesh nebulization, producing a respirable aerosol, and so can be delivered by inhalation to the lung to treat an infection [81]. The nebulized liposomal ciprofloxacin formulation had minimal changes in drug encapsulation, vesicle size, or drug release profile in an IVR assay [81]. Cryo-TEM images before and after mesh nebulization showed that the liposomes retained their spherical structure and unilamellar bilayer [81].

**Figure 17:**
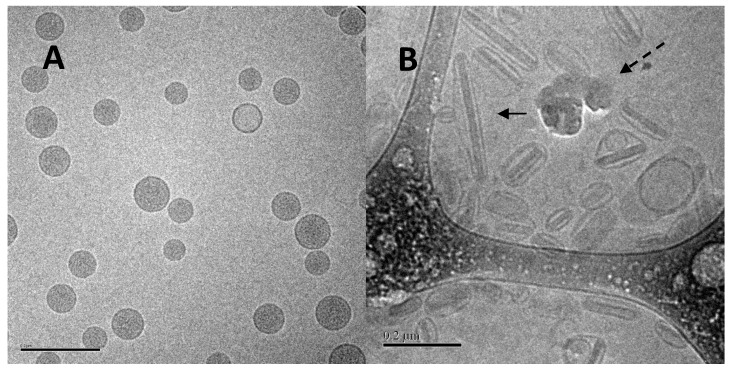
Cryo-TEM micrographs of 12.5 mg/mL Lipoquin, containing 90 mg/mL sucrose, pH 6.0. (**A**) Before freeze-thaw; (**B**) after freeze-thaw. The solid arrow in (**B**) shows a liposome containing a ciprofloxacin nanocrystal. The dashed arrow in (**B**) indicates ice artifacts introduced during sample preparation. Reprinted with permission from [80].

**Figure 18:**
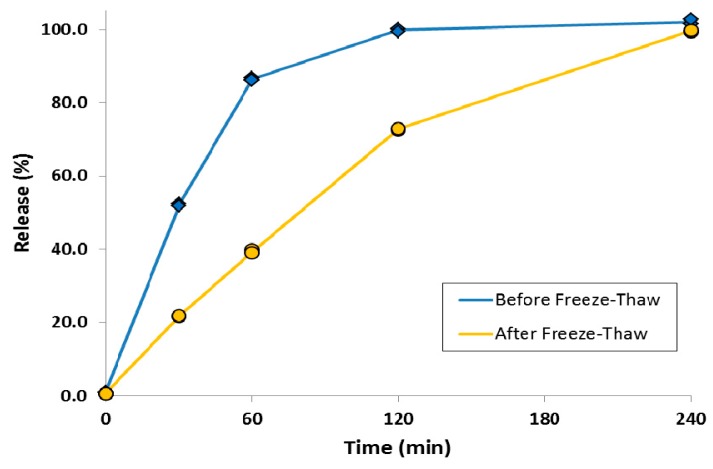
The effect of freeze-thaw process on the *in vitro* release profiles of ciprofloxacin from Lipoquin. Duplicate samples were analyzed at each time point. The IVR assay is described in [43]. Reprinted with permission from [80].

A simple method was also developed to attenuate the release rate of a liposomal ciprofloxacin formulation without changing the lipid composition [80]. This was achieved by converting the drug contained within the liposome vesicles into nanocrystalline form through a single freeze-thaw step (Figure 17). Addition of cryoprotectant (e.g., sucrose) to the liposome formulation was required to stabilize the liposomes to freeze-thaw; this could be accomplished simply by diluting Lipoquin with a concentrated sucrose buffer. After freeze-thaw, the majority of vesicles contained a single drug nanocrystal within the vesicle interior as confirmed by cryo-TEM imaging (Figure 17) [80]. The presence of sucrose internal to the liposome vesicles resulted in shorter nanocrystals (data not shown) [80]. The liposomal formulations containing drug nanocrystals also possessed an attenuated release profile in an *in vitro* release assay [80]. The initial rate of drug release was reduced by ~50%, consistent with the presence of an additional dissolution barrier prior to transport of dissolved drug out of the liposomes (Figure 18) [80].

**Figure 19:**
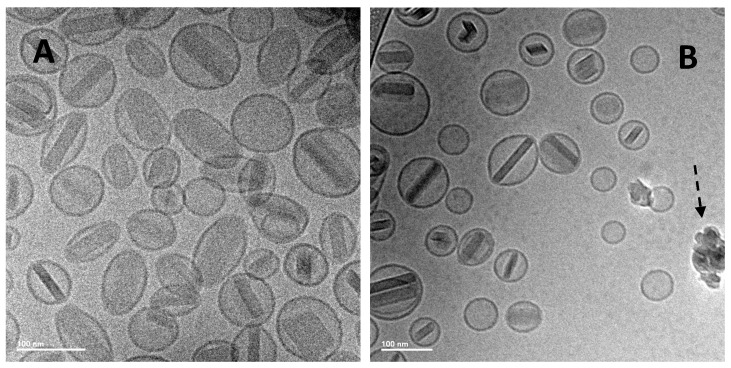
Cryo-TEM micrographs of 12.5 mg/mL Lipoquin, 90 mg/mL sucrose, 0.1% polysorbate 20, pH 5.3. (**A**) After freeze-thaw; (**B**) after freeze-thawing and nebulization. The scale bar in the bottom left-hand corner is 100 nm for both images. The dashed arrow in (**B**) shows ice artifacts introduced during sample preparation. Reprinted with permission from [82].

While the previous liposomal ciprofloxacin formulations, including Lipoquin and Pulmaquin, were successfully aerosolized retaining liposome integrity [41,42,81], it was unclear whether the presence of the ciprofloxacin nanocrystals in the liposomes would reduce their mechanical stability in response to the nebulization process. The potential to aerosolize the liposomes containing ciprofloxacin nanocrystals was thus evaluated using a mesh nebulizer [82]. A respirable aerosol was produced with a volume mean diameter (VMD) between 3.6 and 4.0 µm and a geometric standard deviation (GSD) of 1.7 [82]. The nebulized liposome formulation retained its slow release profile in an IVR assay [82] (data not shown). There was a small increase in vesicle size and a small decrease in drug encapsulation after nebulization [82] (data not shown). Cryo-TEM imaging revealed the presence of intact liposomes containing ciprofloxacin nanocrystals after nebulization, although there may have been a slight increase in the percentage of empty liposomes explaining the slight drop in drug encapsulation (Figure 19) [82].

The long term safety of every inhaled product must be assessed both in preclinical animal toxicology studies as well as in clinical trials. Some of these modified formulations contain a nonionic surfactant (e.g., polysorbate 20). The lung is bathed in lung surfactant composed of proteins, cholesterol and lipids [30,32,83]. A number of synthetic or natural “surfactants” are approved for administration to the lungs to treat respiratory distress syndrome including Exosurf^®^, Curosurf^®^, Infasurf^®^, Survanta^®^ and Surfaxin^®^ [32]. Surfactants are present in marketed metered dose inhaler (MDI) products, e.g., oleic acid, PVP, lecithin, and sorbitan oleates. Surfactants are also used in nebulized suspension products.

In summary, there are now simple methods that can be used to modify the drug encapsulation state, and modify the kinetics of drug release from a liposomal ciprofloxacin formulation. Using these principles, it may one day be possible to personalize therapy to patients with lung infections to improve treatment outcomes.

## 6. Conclusions

The pinnacle of the development of a pharmaceutical product is when it enters clinical evaluation and demonstrates a meaningful benefit to patients. Clinical achievement is predicated upon the successful execution of many activities including formulation, analytical and manufacturing development and preclinical research. For a liposomal formulation, these activities take on even more importance as the liposome provides functional attributes that affect the disposition of the drug substance and these functional attributes must be designed appropriately into the formulation. Furthermore, for an inhaled product, not only must the formulation retain these properties for the duration of its shelf-life, it must also be stable in the aerosolization process. This paper describes the totality of the development of liposomal formulations of ciprofloxacin (Lipoquin and Pulmaquin) through Phase 2 clinical trials. Pulmaquin is now being evaluated in Phase 3 clinical trials to treat lung infections with PA in patients with NCFB and the results are expected towards the end of 2016.

## Figures and Tables

**Table 1:** The aerosol integrity of the first GMP batch of Lipoquin was verified over two-year refrigerated storage and six-month room temperature storage. Reprinted with permission from [41]. Copyright 2010 RDD online.

Storage Condition	Time Point (Months)	Nebulizer		Collected Aerosol		Mass Balance (%)
		Recovery ± SD (%)	Encapsulation ± SD (%)	Emitted Dose ± SD (%)	Encapsulation ± SD (%)	
Release Data	Initial	25.6 ± 1.9	98.8 ± 0.20	67.5 ± 2.8	96.8 ± 0.20	93.1
5 °C	3	23.0 ± 3.9	98.9 ± 0.14	70.7 ± 1.0	96.6 ± 0.10	93.7
	6	20.9 ± 0.6	99.0 ± 0.04	74.0 ± 1.7	98.1 ± 0.16	94.9
	9	26.5 ± 1.0	99.2 ± 0.01	65.9 ± 0.9	98.7 ± 0.02	91.5
	12	23.8 ± 1.2	98.9 ± 0.08	66.0 ± 1.9	97.7 ± 0.17	89.8
	18	23.8 ± 0.7	99.0 ± 0.09	71.8 ± 2.1	98.6 ± 0.21	95.6
	24	24.5 ± 0.8	99.2 ± 0.06	66.0 ± 1.2	97.6 ± 0.02	90.5
25 °C	3	25.0 ± 2.5	99.1 ± 0.05	69.4 ± 1.4	96.7 ± 0.21	94.4
	6	21.9 ± 3.0	98.7 ± 0.16	72. 6± 1.4	96.9 ± 0.49	93.3

**Table 2:** GMP batches of Lipoquin retain integrity after 24-month storage at refrigerated conditions and subsequent nebulization. Adapted with permission from [41]. Copyright 2010 RDD online.

Lot Number	Control		Nebulizer			Collected Aerosol			Mass Balance (%)
	Encapsulation ± SD (%)	Mean Size (nm)	Recovery ± SD (%)	Encapsulation ± SD (%)	Mean Size (nm)	Emitted Dose ± SD (%)	Encapsulation ± SD (%)	Mean Size (nm)	
Lot 4	99.6 ± 0.1	89.4	41.8 ± 1.6	99.4 ± 0.2	92.7	53.3 ± 1.1	96.0 ± 0.1	89.9	95.1
Lot 5	99.6 ± 0.1	90.6	39.8 ± 2.3	99.5 ± 0.0	90.7	56.0 ± 0.6	96.4 ± 0.2	91.7	95.8
Lot 6	99.5 ± 0.1	86.2	39.5 ± 0.2	99.4 ± 0.1	85.6	54.6 ± 1.9	97.0 ± 0.4	85.7	94.1
Lot 7	99.6 ± 0.0	90.4	44.0 ± 0.2	99.6 ± 0.0	96.1	51.9 ± 0.9	96.9 ± 0.9	93.0	95.9
Lot 8	97.0 ± 2.5	74.7	34.1 ± 2.3	98.5 ± 1.1	83.6	60.5 ± 1.3	96.1 ± 2.3	83.8	94.6
Lot 9	99.6 ± 0.1	89.3	42.0 ± 2.5	99.4 ± 0.2	88.2	53.9 ± 1.3	96.5 ± 0.9	91.3	95.9
Lot 10	99.6 ± 0.0	83.8	42.0 ± 0.3	99.6 ± 0.0	86.2	52.3 ± 1.1	97.1 ± 0.4	88.8	94.3
Lot 11	99.5 ± 0.1	86.8	40.3 ± 4.6	99.6 ± 0.0	88.0	52.3 ± 1.9	97.0 ± 0.1	not tested	92.6
Lot 12	98.8 ± 1.0	91.5	40.6 ± 1.6	99.6 ± 0.1	90.5	55.4 ± 1.7	97.2 ± 0.1	not tested	96.0
Mean	99.2 ± 0.9	87.0 ± 5.2	40.5 ± 2.8	99.4 ± 0.4	89.1 ± 3.9	54.5 ± 2.7	96.7 ± 0.5	89.2 ± 3.3	94.9 ± 1.1

## Supplementary Materials

The following are available online at www.mdpi.com/1999-4923/8/1/6/s1, Additional methodological details for Section 3.2, Section 3.3 and Section 3.4.1; Table S1: Scoring clinical signs in mice; Table S2: Survival in CF mice with PA lung infection treated with Lipoquin or control; Table S3: Survival in CF Mice with PA Lung Infection Treated with Lipoquin, “Pulmaquin-prototype” or Control; Table S4: Survival in CF Mice with PA Lung Infection Treated with TOBI, Lipoquin, “Pulmaquin-prototype” or Control; Figure S1: Therapeutic efficacies of oral ciprofloxacin and intranasal Lipoquin against inhalational *F. tularensis* Schu S4 infection in mice.

## Abbreviations

The following abbreviations are used in this manuscript:

AUC: Area under the curve
BIC: biofilm inhibitory concentration
BID: twice daily
CF: cystic fibrosis
CFTR: cystic fibrosis transmembrane conductance regulator
CFU: colony forming unit
*C*_max_: maximum concentration
CFI: ciprofloxacin for inhalation (Lipoquin, liposomal ciprofloxacin)
CH: cholesterol
CIP: ciprofloxacin
COPD: Chronic Obstructive Pulmonary Disease
Cryo-TEM: cryogenic transmission electron microscope
D25: diameter for which 25% of the particles have a smaller size
D50: diameter for which 50% of the particles have a smaller size
D75: diameter for which 75% of the particles have a smaller size
D90: diameter for which 90% of the particles have a smaller size
DOX: Doxycycline
DLS: dynamic light scattering
EPC: egg phosphatidylcholine
ESM: egg sphingomyelin
FCI: free ciprofloxacin for inhalation (unencapsulated ciprofloxacin)
FEV_1_: forced expiratory volume in one second
GLP: good laboratory practices
GMP: good manufacturing processes
GSD: geometric standard deviation
HSPC: hydrogenated soy phosphatidylcholine
IV: intravenous
IVR: *In Vitro* Release
LB: lysogeny broth
LPS: lipopolysaccharide
MDI: metered dose inhaler
MIC: minimum inhibitory concentration
mITT: modified intention to treat
NA: not applicable
NCFB: non-cystic fibrosis bronchiectasis
NTM: non-tuberculous mycobacteria
OD: optical density
ORBIT: Once-daily Respiratory Bronchiectasis Inhalation Treatment
PA: Pseudomonas aeruginosa
PBS: phosphate buffered saline
PC: phosphatidylcholine
PEP: post-exposure prophylaxis
POPC: 1-palmitoyl-2-oleoylphosphatidylcholine
Pulm: pulmonary
QoL: Quality of Life
SAE: serious adverse event
VMD: volume mean diameter
