# Supplementary material for: Development of Liposomal Ciprofloxacin to Treat Lung Infections
Source: Pharmaceutics. 2016 Mar 1;8(1):6. doi: 10.3390/pharmaceutics8010006 (PMC4810082; doi:10.3390/pharmaceutics8010006)
Supplement: Supplementary file 1 [file pharmaceutics-08-00006-s001.pdf]

Supplementary Materials: Development of Liposomal Ciprofloxacin to Treat Lung Infections

David Cipolla *, Jim Blanchard and Igor Gonda

Additional methodological details as well as data are provided for the following sections:

3.2. Efficacy against PA: In Vitro Studies

The isolates were cultured overnight to form a diluted culture, which was then incubated in LB medium for 24 h in a 96-well microtiter plate. Negative controls were cell culture medium, saline buffer and empty liposomes. The lid of the microtiter plate was modified so the pegs were immersed in the diluted culture and thereby provided a surface for biofilm formation. Next, the biofilms were challenged with Lipoquin, "Pulmaquin-prototype", free ciprofloxacin, or controls: LB medium control, buffer control and empty liposome control.

The challenge was done by first transferring each peg lid to a microtiter plate with the formulations at doubling concentrations (0.25–32 μg/mL) and then incubating for 24 h. Next, the peg lids were transferred to antibiotic-free LB medium and then centrifuged to release the biofilms from the pegs into the medium in the microtiter plate. The resulting biofilm biomass was based on the optical density at 650 nm (OD650) in the LB medium measured before and after incubation for 6 h, such that low OD values reflect biofilm killing. Adequate biofilm growth over the 6 h for the positive control wells was defined as a mean difference in OD650 of greater than 0.05 between the OD650 at $t = 0$ and $t = 6$ h.

3.3. Efficacy against PA: In Vivo Studies

The mice were gut-corrected, *Cftr*-knockout mice that had a mixed genetic background. The mice (9 to 10 per sex, per group, or per study) were inoculated with PA-laden agarose beads instilled into the lungs on Day 0, and the infection was allowed to develop for two days before treatment started. The PA dose was targeted to be lethal in 50%–75% of the control mice so a treatment effect could be observed. Mice received once- or twice-daily intranasal instillations of Lipoquin or control solutions, starting on Day 2 and ending on Day 9. Negative controls received diluent in the same manner. Surviving mice were sacrificed on Day 10.

The system for scoring clinical signs is shown in Table A1:

Table A1. Scoring clinical signs in mice.

Clinical Score	Clinical Signs
0	Healthy appearance, normal activity
1	Slightly scruffy or scruffy appearance
2	Slightly scruffy and slightly dehydrated, or scruffy and slightly dehydrated
3	Scruffy, dehydrated, decreased activity
4	Scruffy, dehydrated, decreased activity, hunched back, slow gait
5	Moribund (severe delay in the righting reflex and palpably cold; mice are euthanized for humane reasons) or dead

In the first study, mice were treated daily with Lipoquin at estimated lung doses of 0.2, 0.4, or 0.9 mg/kg, which are equivalent to clinical lung doses of 10, 20, and 45 mg in a 50-kg patient. The results indicate treatment with Lipoquin increases survival time and improves clinical signs in CF mice (Table A2). The 0.9 mg/kg group had 90% survival, whereas the lower doses and controls had survivals ranging from 45%–67%. Although the survival rates among the groups did not differ significantly from one another, mice treated with Lipoquin were associated with higher survival rates and improved clinical signs compared to mice treated with diluent.

Table A2. Survival in CF mice with PA lung infection treated with Lipoquin or control.

Lipoquin Instilled Dose (mg)	Lipoquin Lung Dose (mg/kg) *	*N* on Day 2	*N* on Day 10	Mortality	Survival Rate Day 2 to Day 10	Clinical Score on Day 10
Diluent Control	0	9	4	5/9	44% (4/9)	3.11 ± 2.32
0.025	0.2	9	6	3/9	67% (6/9)	2.00 ± 2.35
0.050	0.4	9	4	5/9	44% (4/9)	3.22 ± 2.28
0.125	0.9	10	9	1/10	90% (9/10)	1.40 ± 1.78

* Ciprofloxacin lung dose = instilled dose × 15% delivery efficiency to lungs / mouse body weight (assumed 20 g).

In subsequent studies replicated 3 times, the efficacy of both Lipoquin and a "Pulmaquin-prototype" formulation (1:1 *v*/*v* 50 mg/mL Lipoquin and 30 mg/mL FCI) was evaluated (Table A3). The "Pulmaquin-prototype" lung dose of 0.96 mg/kg had 65% survival. The Lipoquin lung doses of 0.6 and 0.98–1.2 mg/kg had 55%–70% survival. In contrast, the control (diluent) had 43% survival. Therefore, the mice treated with "Pulmaquin-prototype" and Lipoquin had higher survival rates and better clinical scores compared to mice treated with diluent; however, these results were not statistically significant.

Table A3. Survival in CF Mice with PA Lung Infection Treated with Lipoquin, "Pulmaquin-prototype" or Control.

Treatment	Ciprofloxacin Instilled Dose (mg)	Ciprofloxacin Lung Dose (mg/kg) *	*N* on Day 2	*N* on Day 9	Total Mortality	Survival Rate Day 2 to Day 10	Clinical Score on Day 10
Control (Diluent)	0	0	28	12	16/28	43% (12/28)	3.07 ± 2.39
Lipoquin-Low	0.08	0.60	27	19	8/27	70% (19/27)	1.81 ± 2.29
Lipoquin-High	0.13–0.16 §	0.98–1.2 §	27	15	12/27	55% (15/27)	2.48 ± 2.47
"Pulmaquin"	0.128	0.96	31	20	11/31	65% (20/31)	2.06 ± 2.38

* Ciprofloxacin lung dose = instilled dose × 15% delivery efficiency to lungs / mouse body weight (assumed 20 g); § Range of doses pooled from three studies.

Studies were also conducted to compare the efficacy of Lipoquin versus tobramycin (TOBI®, Novartis). The control group was instilled with diluent. The study design is shown in Table A4. For each treatment, the TOBI group received a lung dose of 11 mg/kg, which represented 25 μL of the standard 60 mg/mL formulation delivered with 15% efficiency in a 20-g mouse. Since the standard of care is to administer TOBI twice daily (BID), the TOBI group received two treatments per day: one in the morning and the second 12 h later in the evening. To control for the evening treatment, the other groups received an air sham instillation. Surviving mice were sacrificed on Day 10.

Table A4. Survival in CF Mice with PA Lung Infection Treated with TOBI, Lipoquin, "Pulmaquin-prototype" or Control.

Treatment	Instilled Dose (mg)	Lung Dose (mg/kg) *	*N* on Day 2	*N* on Day 10	Mean Mortality	Mean Survival (%)	Clinical Score on Day 10
Control	0	0	10	4	6/10	40% (4/10)	3.70 ± 1.77
TOBI	3.0 (daily)	22 (daily)	10	4	6/10	40% (4/10)	3.70 ± 2.06
Lipoquin	0.16	1.2	10	9	1/10	90% (9/10)	1.40 ± 1.71
"Pulmaquin"	0.128	0.96	9	6	3/9	67% (6/9)	2.33 ± 2.35

* Ciprofloxacin lung dose = instilled dose × 15% delivery efficiency to lungs / mouse body weight (assumed 20 g).

3.4.1. Pneumonic (Respiratory/Inhalational) Tularemia–Lipoquin Efficacy against *F. tularensis*

Female BALB/c mice were challenged with a lethal dose (about 10 CFU) of aerosolized *F. tularensis* Schu S4. The infection was allowed to develop for 24 h and then therapy began, which consisted of intranasally instilled Lipoquin (50 mg/kg once daily), aerosolized Lipoquin (1 mg/kg lung dose delivered once daily), or oral ciprofloxacin (50 mg/kg either once or twice daily). Three dosing regimens were tested: single dose on one day or for 3 or 5 days, with 12 animals per group. The clinical signs of the mice were monitored twice-daily for 28 days post-challenge.

When treatment was extended to 3 days, once daily intranasal Lipoquin provided over 90% survival (Figure 10b) and 5 days provided 100% protection (Figure 10c), and significantly increased time to death ($p < 0.005$) compared to untreated controls. Conversely, twice-daily oral ciprofloxacin provided no protection at 3 days and less than 20% protection at 5 days. Aerosolized Lipoquin was not evaluated at 3 and 5 days but the single dose experiments suggest that aerosolized Lipoquin would likely have even greater efficacy. From a PK perspective, intranasal Lipoquin (50 mg/kg), which has a half-life of 6.6 h, provided over a 5600-fold greater $AUC_{0–24}$ in the lungs than oral ciprofloxacin at the same dose level (50 mg/kg) [50].

A possible explanation for the inferior efficacy of the single dose of intranasal Lipoquin to that of the single dose of aerosol Lipoquin (Figure 10) may be due to the mismatch between the route of administration of the infectious agent (by aerosol) and the therapy (intranasal instillation). Aerosol delivery of either the infectious agent or the therapeutic intervention would result in more uniform deposition throughout the lung in contrast to the intranasal instillation which would result in delivery to a smaller more focal part of the lungs [62]. Since the *F. tularensis* challenge was delivered by aerosol, the pathogen would be delivered uniformly throughout the lungs. Similarly when the Pulmaquin formulation is delivered by aerosol, it also would be delivered uniformly throughout the lungs; therefore, the Pulmaquin is delivered to the essentially the same sites in the lungs as the infection, maximizing the treatment's efficacy. In contrast, drug delivered by intranasal instillation would be delivered to a relatively small focal part of the lung; therefore, it would not be in the same parts of the lung that were infected by aerosol [62]; thus the efficacy may be reduced.

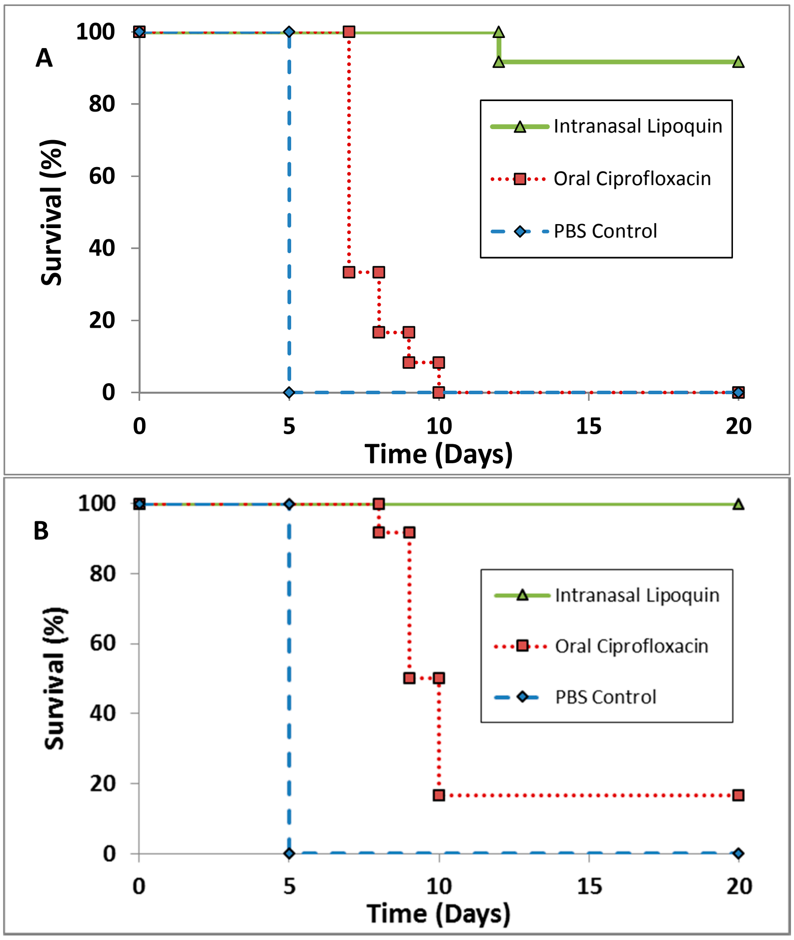

Figure S1. Therapeutic efficacies of oral ciprofloxacin and intranasal Lipoquin against inhalational *F. tularensis* Schu S4 infection in mice. Groups of 12 BALB/c mice were challenged with *F. tularensis* Schu S4 via the aerosol route and treated at 24 h post-challenge with 50 mg/kg of oral ciprofloxacin (red squares, dotted line), 50 mg/kg of intranasal Lipoquin (green triangles, solid lines), or intranasal PBS (blue diamonds, dashed line). Graphs show the survival of mice treated with: (**A**) 3 days of therapy, or (**B**) 5 days of therapy.
